# Ligand-Modulated Nuclearity and Geometry in Nickel(II) Hydrazone Complexes: From Mononuclear Complexes to Acetato- and/or Phenoxido-Bridged Clusters

**DOI:** 10.3390/ijms24031909

**Published:** 2023-01-18

**Authors:** Višnja Vrdoljak, Tomica Hrenar, Mirta Rubčić, Gordana Pavlović, Tomislav Friganović, Marina Cindrić

**Affiliations:** 1 Department of Chemistry, Faculty of Science, University of Zagreb, Horvatovac 102a, 10000 Zagreb, Croatia; 2 Department of Applied Chemistry, Faculty of Textile Technology, University of Zagreb, Prilaz Baruna Filipovića 28a, 10000 Zagreb, Croatia

**Keywords:** 4-hydroxybenzhydrazone, Ni(II) complexes, clusters, hybrid organic–inorganic, polyoxometalate, acetato, phenoxido, bridged complexes, nuclearity

## Abstract

The propensity of 4-hydroxybenzhydrazone-related ligands derived from 3-methoxysalicylaldehyde (H_2_L^3OMe^), 4-methoxysalicylaldehyde (H_2_L^4OMe^), and salicylaldehyde (H_2_L^H^) to act as chelating and/or bridging ligands in Ni(II) complexes was investigated. Three clusters of different nuclearities, [Ni_3_(L^3OMe^)_2_(OAc)_2_(MeOH)_2_]∙2MeOH∙MeCN (**1**∙2MeOH∙MeCN), [Ni_2_(HL^4OMe^)(L^4OMe^)(OAc)(MeOH)_2_]∙4.7MeOH (**2**∙4.7MeOH), and [Ni_4_(HL^H^)_2_(L^H^)_2_(OAc)_2_]∙4MeOH·0.63H_2_O·0.5MeCN·HOAc (**3**∙4MeOH·0.63H_2_O·0.5MeCN·HOAc), were prepared from Ni(OAc)_2_∙4H_2_O and the corresponding ligand in the presence of Et_3_N. The hydrazones in these acetato- and phenoxido-bridged clusters acted as singly or doubly deprotonated ligands. When pyridine was used, mononuclear complexes with the square-planar geometry seemed to be favoured, as found for complexes [Ni(L^3OMe^)(py)] (**4**), [Ni(L^4Ome^)(py)] (**5**) and [Ni(L^H^)(py)] (**6**). Ligand substituent effects and the stability of square-planar complexes were investigated and quantified by extensive quantum chemical analysis. Obtained results showed that standard Gibbs energies of binding were lower for square-planar than for octahedral complexes. Starting from [MoO_2_(L)(EtOH)] complexes as precursors and applying the metal-exchange procedure, the mononuclear complexes [Ni(HL^3OMe^)_2_]∙MeOH (**7**∙MeOH) and [Ni(HL^H^)]∙2MeOH (**9**∙2MeOH) and hybrid organic–inorganic compound [Ni_2_(HL^4OMe^)_2_(CH_3_OH)_4_][Mo_4_O_10_(OCH_3_)_6_] (**10**) were achieved. The octahedral complexes [Ni(HL)_2_] (**7**–**9**) can also be obtained by the direct synthesis from Ni(Oac)_2_∙4H_2_O and the appropriate ligand under specific reaction conditions. Crystal and molecular structures of **1**∙2MeOH∙MeCN, **2**∙4.7MeOH, **3**∙4MeOH∙0.63H_2_O∙0.5MeCN∙HOAc, **4**, **5**, **9**∙2MeOH, and **10** were determined by the single-crystal X-ray diffraction method.

## 1. Introduction

The chemistry of multinuclear coordination compounds has been receiving ongoing interest because of their unique and attractive structures, interesting properties, and potential applications such as magnetic materials, solar energy conversion systems, and electroluminescent and photovoltaic devices [1,2,3,4,5,6,7,8,9]. Investigations span the whole range of research fields, from theoretical to biomimetic [10,11]. To date, many research groups are attempting to synthesise Ni(II) complexes bridged by carboxylate and hydroxido groups to mimic the structure and catalytic function of the active site of the native urease enzymes [12,13,14,15,16]. 

The formation of a specific multinuclear architecture largely relies on the choice of ligand(s) and/or reaction conditions. Thus, bridging ligands containing at least one deprotonated *O*-donor atom (hydroxo, alkoxo, oximato, etc.) have been successfully used for their synthesis [17,18]. Aroylhydrazone ligands ArC=N–NH–(C=O)–R with *O*-donor atoms have the ability to bridge metallic ions and to build a range of polymetallic arrays [19,20,21]. We have reported the coordination properties of benzhydrazone-related ligands, and conditions for obtaining a number of dinuclear, cubane tetranuclear, and polynuclear copper(II) complexes [22,23].

The protonation state of these ligands in metal complexes plays an important role as it offers fine-tuning of properties [24,25,26]. The ability of aroylhydrazones to act as neutral or as anionic ligands (in singly deprotonated HL^−^ or doubly-deprotonated forms L^2–^; Scheme S1) allows them to form a diverse range of metal complexes [27,28,29,30,31,32,33,34,35]. Some examples of structurally characterised nickel(II) complexes with hydrazone ligands include [Ni{dap(A)_2_}]_2_ (dap(AH)_2_ = 2,6-diacetylpyridine bis(anthraniloyl hydrazone) [36], [Ni(H_3_bsc)_2_](ClO_4_)_2_ (H_3_bsc = 1,5-bis(salicylidene)carbohydrazide) [37], [Ni(L)(PPh_3_)] (L = hydroxylbenzylidenebenzenecarbohydrazone) [38], [Ni(L)]_2_ (L = ferrocenyl hydrazone ligands [39], and [NiL_2_phen]·CH_3_CN (HL = 2-acetonaphthone salicylhydrazone, phen = 1,10-phenanthroline) [40].

Although numerous complexes with aroylhydrazone derivatives are known, related nickel(II) complexes based on differently deprotonated ligands have not been reported so far. Generally, these complexes are very scarce, and the structures of [Fe{H(3,5-*^t^*Bu_2_)salbh}{(3,5-*^t^*Bu_2_)salbh}] [41], [Fe(HL)(L)] [42], and [Co(HL^1–6^)(L^1–6^)] [43] represent such examples. Their formation is followed simultaneously by the hydrazonato-hydrazidato tautomeric maneouver of the ligands (Scheme S1) [41].

**Scheme 1:**
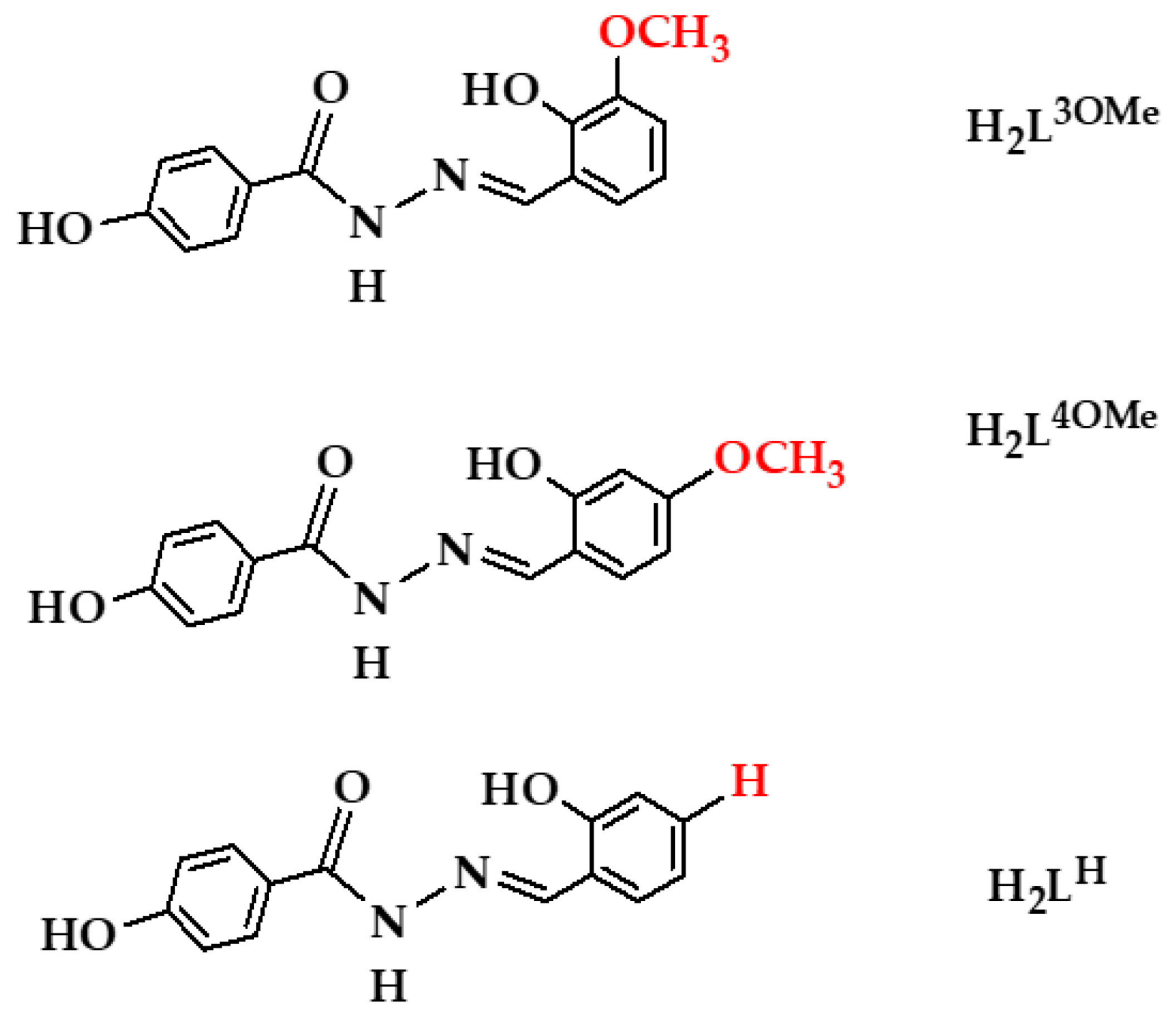
The 4-hydroxybenzhydrazone-related ligands H_2_L (H_2_L^3OMe^, H_2_L^4OMe^, and H_2_L^H^). The substituent is shown in red.

Inspired by the previously mentioned facts, we aimed to investigate: (a) *ONO* chelating and *O*-bridging capabilities of ligands; (b) synthetic conditions under which nickel(II) complexes of different nuclearities and geometries can be obtained; (c) factors that may be used to control the nuclearity of clusters; (d) effects of ligand substituents on the binding modes of a hydrazone ligand. To achieve these aims, we chose three ligands, (3-methoxysalicylaldehyde 4-hydroxybenzhydrazone (H_2_L^3OMe^), 4-methoxysalicylaldehyde 4-hydroxybenzhydrazone (H_2_L^4OMe^), and salicylaldehyde 4-hydroxybenzhydrazone (H_2_L^H^); Scheme 1). Understanding these factors will certainly add insight into different aspects of the multinuclear system assembly process.

## 2. Results and Discussion

The appropriate synthetic approach, i.e., the stoichiometric ratio of Ni(II) salt and 4-hydroxybenzhydrazone-related starting compounds, the addition of triethylamine or pyridine, adequate reaction temperature, and other conditions, allowed the formation of three acetato- and phenoxido-bridged di-, tri-, and tetranuclear clusters, six mononuclear complexes, and one hybrid organic–inorganic compound (Scheme S2). It is important to mention that multinuclear clusters bearing tridentate *ONO* hydrazone ligands and acetato-bridging ligands have not been structurally characterised previously. Only examples of trinuclear acetato-bridged nickel clusters containing tridentate Schiff base ligands have been reported so far [44,45,46,47].

### 2.1. Acetato- and Phenoxido-Bridged Di-, Tri-, and Tetranuclear Nickel(II) Clusters

**Scheme 2:**
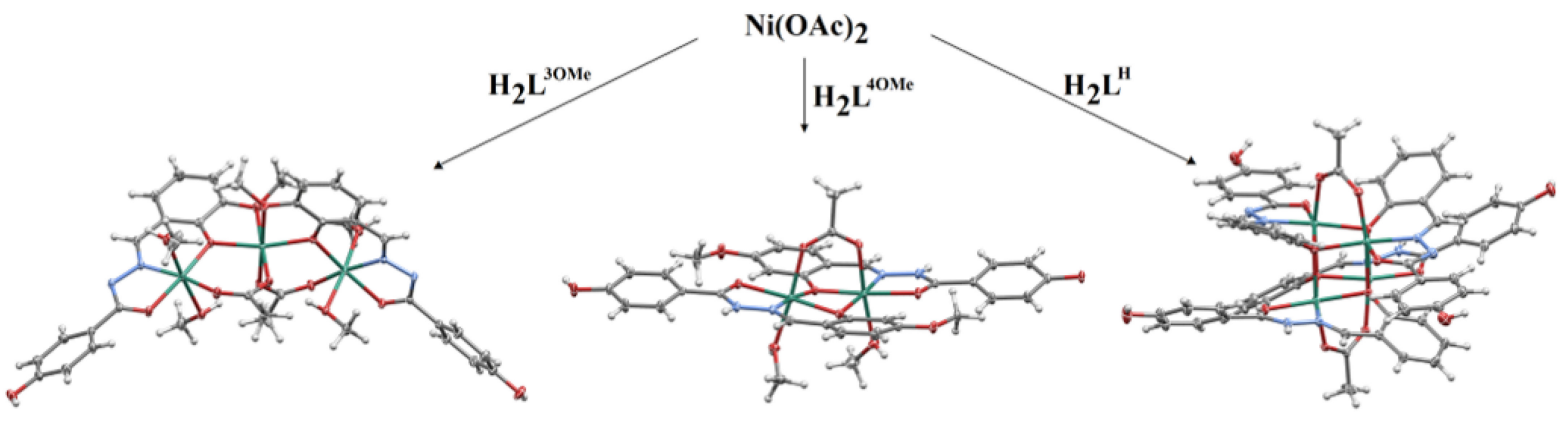
Reaction pathways and structures of **1**∙2MeOH∙MeCN, **2**∙4.7MeOH, and **3**∙4MeOH∙0.63H_2_O∙0.5MeCN∙HOAc. All reactions were performed in the presence of Et_3_N in a methanol and acetonitrile mixture. The ligands H_2_L^3OMe^, H_2_L^4OMe^, or H_2_L^H^ are depicted in Scheme 1. Color code: Ni-green, N-blue, O-red, C-gray, H-white.

Structurally diverse complexes were prepared by the reaction of Ni(OAc)_2_∙4H_2_O with a stoichiometric amount of the corresponding aroylhydrazone ligand H_2_L^3OMe^∙H_2_O, H_2_L^4OMe^∙H_2_O, or H_2_L^H^ (Scheme 2). All reactions were performed in the presence of triethylamine in a methanol and acetonitrile mixture. Very delicate and unstable crystals of trinuclear [Ni_3_(L^3OMe^)_2_(Oac)_2_(MeOH)_2_]∙2MeOH∙MeCN (**1**∙2MeOH∙MeCN), dinuclear [Ni_2_(HL^4OMe^)(L^4OMe^)(Oac)(MeOH)_2_]∙4.7MeOH (**2**∙4.7MeOH), and tetranuclear [Ni_4_(HL^H^)_2_(L^H^)_2_(Oac)_2_]∙4MeOH∙0.63H_2_O∙0.5MeCN∙HOAc (**3**∙4MeOH∙0.63H_2_O∙0.5MeCN ∙HOAc) clusters were harvested directly from the reaction mixture, and these were suitable for the single-crystal X-ray diffraction experiments. The analysis revealed a rapid solvent loss at room temperature. A sample of **3,** therefore, showed significant loss of crystallinity, whereas under the same conditions, **1** and **2** turned into amorphous solids. 

The bridging ligands, such as acetate, are crucial for the construction of the clusters. However, the hydrazone ligand with phenoxido-bridges also plays an important role in maintaining the cluster structure. The assembly process is additionally sensitive to the position of the methoxy group of the ligand. Namely, in cluster **1**∙2MeOH∙MeCN derived from L^3OMe^, it is sterically in an almost perfect position to participate in complexation. The results also indicate a marked relationship between the nuclearity of Ni(II) clusters and the tautomeric form of the deprotonated ligand (Scheme S1). Thus, H_2_L^3OMe^ undergoes complexation via tautomerisation of the keto-form, double-deprotonation, and formation of the trinuclear cluster **1**∙2MeOH∙MeCN. In contrast, ligands H_2_L^4OMe^ and H_2_L^H^ participate in complexation via differently deprotonated forms, HL^−^ and L^2−^ forms, thus yielding dinuclear and tetranuclear complexes, **2**∙4.7MeOH and **3**∙4MeOH∙0.63H_2_O∙0.5MeCN∙HOAc, respectively. The presence of hydrazidato and hydrazonato tautomeric forms of the ligand (having =N–NH–(C=O)– and =N–N=(C–O^−^)– moiety, respectively) is supported by IR spectroscopy as well as by the single-crystal X-ray diffraction method. The occurrence of the different forms can be explained by the electron donor effect of the methoxy group at the ortho position, which may promote ligand tautomerisation and deprotonation during the complexation. 

#### 2.1.1. Structure of [Ni_3_(L^3OMe^)_2_(OAc)_2_(MeOH)_2_]∙2MeOH∙MeCN 

**Figure 1:**
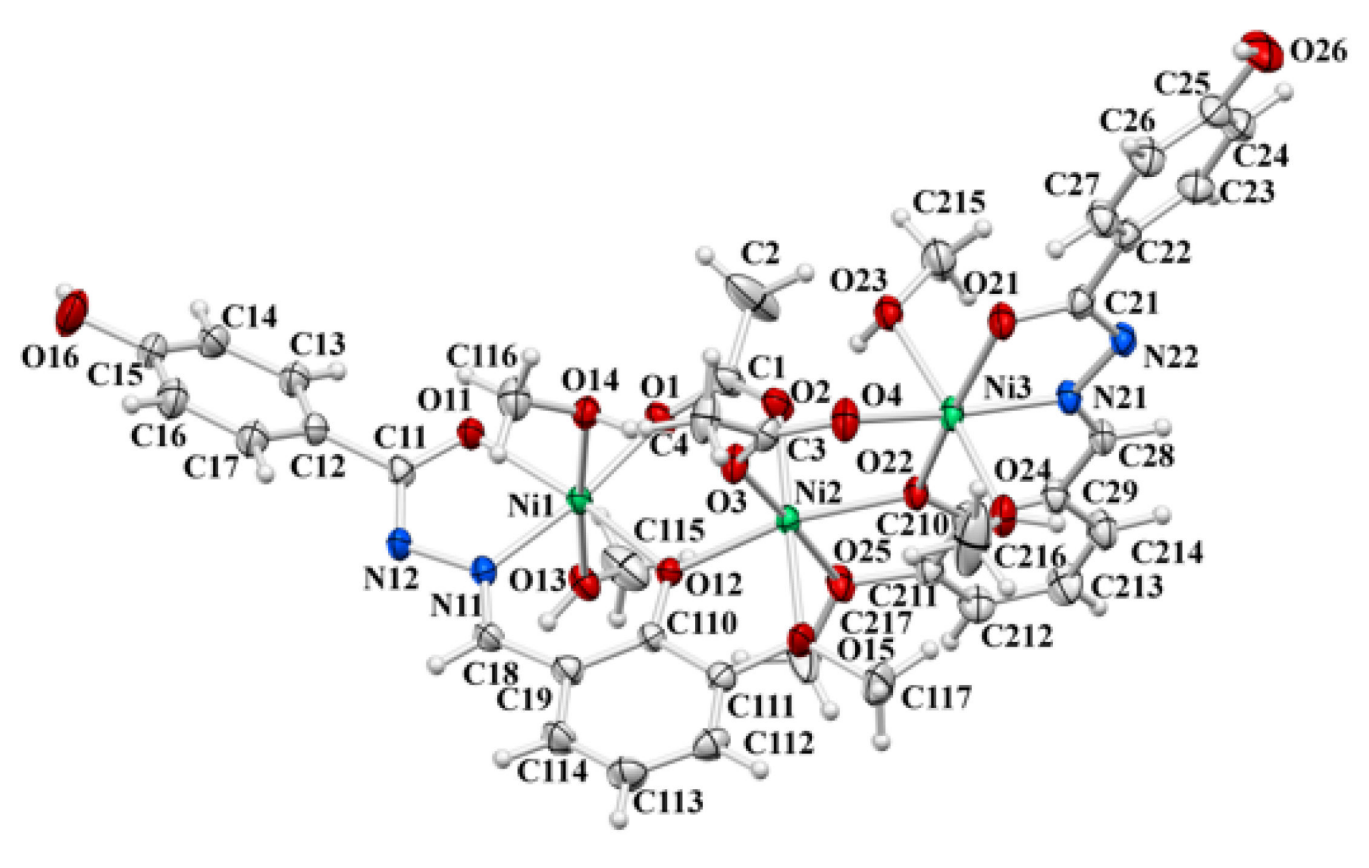
ORTEP view of molecular structure of **1**∙2MeOH∙MeCN. The displacement ellipsoids are drawn at the 50% probability level at 150(2) K. The solvent molecules of crystallisation have been omitted for clarity.

The molecular structure of the trinuclear complex **1**∙2MeOH∙MeCN (Figure 1) reveals three octahedrally coordinated Ni(II) ions. The Ni1 and Ni3 atoms are coordinated by NO5 donor atoms set in an analogous manner, while the central Ni2 atom is coordinated by six oxygen atoms. The coordination sphere of Ni1 and Ni3 is built up of two hydrazonato L^2−^ ligands, one oxygen atom from the acetate ion, and two methanol molecules in apical positions of an octahedron. The hydrazonato ligand surrounds peripheral Ni1 and Ni3 ions, forming two chelate rings fused along Ni1–N11 and Ni3–N21 bonds. 

The central Ni2 atom is surrounded by two bridging phenoxido atoms O12 and O22, of two hydrazone ligands, two hydrazone methoxy O15 and O25 atoms, and the O2 and O3 atoms from two acetate ions. The methoxy and bridging phenoxido donor atoms form a five-membered chelate ring at Ni2. The bond distances values within coordination spheres (Table S1) are between 1.990(3) Å (for Ni3–N21 bond distance) and 2.169 (3) Å (for Ni3–O23 bond distance with coordinated methanol molecule), being slightly longer than in mononuclear octahedral Ni complexes **4** and **5**. These coordination modes govern Ni…Ni separations of 3.486(1) Å and 3.468(1) Å for Ni1…Ni2 and Ni2…Ni3 separations, respectively, spanning nickel centres in the bent fashion (Ni1–Ni2–Ni3 angle amounts 144.76(2)°).

The hydrazonato L^2−^ ligand form is evidenced by the C–O single-bond length values of the five-membered chelate ring: C11–O11 and C21–O21 of 1.296(4) and 1.289(4) Å, respectively, which are most susceptible on ligand tautomerisation. Although the methoxy group does not exhibit remarkable donor capabilities, especially in comparison with the imino nitrogen or phenoxido oxygen atoms for the metal coordination sphere, its position on the aldehyde residue (ring) allows it to participate in coordination. 

**Figure 2:**
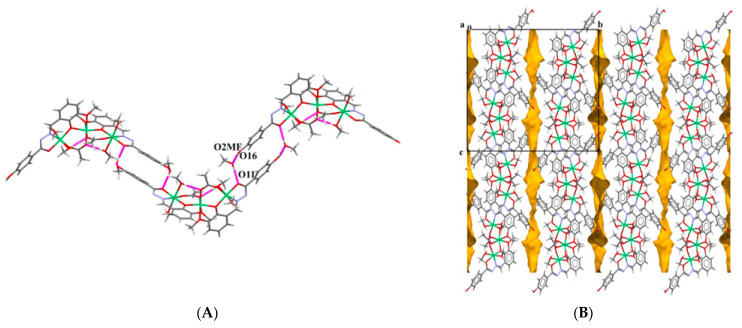
(**A**) Crystal packing view of **1**∙2MeOH∙MeCN showing assembly of complex molecules into infinite chains of *R*$\frac{4}{4}$(20) centrosymmetric rings via two hydrogen bonds: O16–H16O···O2ME and O2ME–H2ME···O11. (**B**) The solvent molecules have been omitted and voids are denoted in yellow forming channels. Color code: Ni-green, N-blue, O-red, C-gray, H-white.

Intramolecular hydrogen bonds between coordinated methanol molecules and the acetate O3 and O2 atoms, O14–H14O···O3 and O23–H23O···O2, span three nickel centres. The complex molecules are assembled into infinite chains of *R*$\frac{4}{4}$(20) centrosymmetric rings via O16–H16O···O2ME and O2ME–H2ME···O11 hydrogen bonds (O2ME–H2ME denotes O–H functionality of the non-coordinated methanol molecule) (Figure 2A). Many C–H···O-type hydrogen bonds snap complex molecules, forming crystal structures. The acetonitrile molecules are loosely bound to a trinuclear cluster by means of the C–H···N weak hydrogen bonds acting as a proton acceptor. The proton donors are the aryl –CH group and less acidic methyl C217–H217B group (Table S2). The solvent molecules are accommodated within channels formed between complex molecules in an AB alternating fashion along the *b* axis (Figure 2B). 

#### 2.1.2. Structure of [Ni_2_(HL^4OMe^)(L^4OMe^)(OAc)(MeOH)_2_]∙4.7MeOH

**Figure 3:**
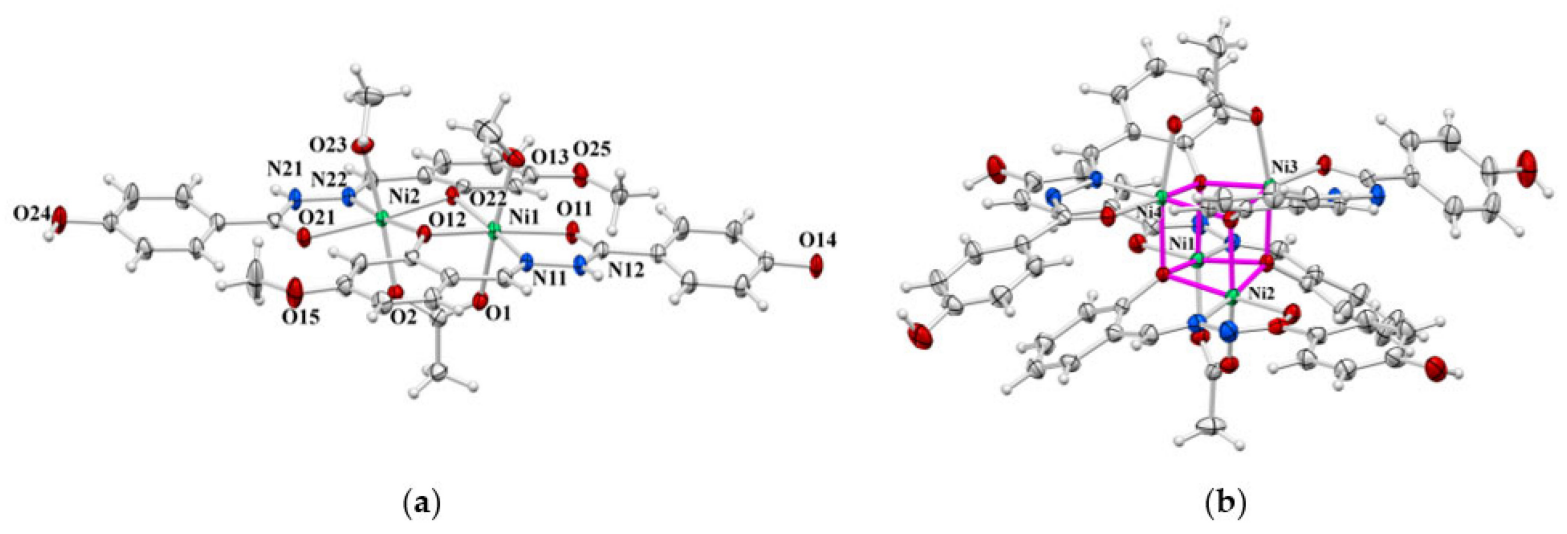
ORTEP view of molecular structures of: (**a**) **2∙**4.7MeOH and (**b**) **3**∙MeOH∙0.63H_2_O ∙0.5MeCN∙HOAc. The displacement ellipsoids are drawn at the 50% probability level at 150(2) K. The solvent molecules of crystallisation have been omitted.

The asymmetric unit of **2**∙4.7MeOH (Figure 3a) contains two Ni ions, two hydrazidato ligands, one acetate ion, two coordinated methanol molecules, and 4.7 methanol molecules of crystallisation. Each Ni ion is octahedrally coordinated by a NO5 donor atoms set formed by a tridentate *ONO* hydrazidato ligand, bridging acetate ion, and the methanol molecule. The phenoxido O12 and O22 donor atoms act as bridging ones, thus forming a Ni2O2 non-centrosymmetric moiety with a Ni1…Ni2 separation of 3.004(1) Å. The shortest bond distances are Ni–N in relation to Ni–O, which are supposed to be longer than 2.00 Å for an octahedral Ni coordination environment (Table S3). Despite the bridging function, the Ni–O bond distances are not longer than in mononuclear octahedral Ni complexes. The deviation from the regular nickel octahedral surrounding is described by *trans* bond angle values formed by atoms in apical positions: O1–Ni1–O13 174.27(7)° and O2–Ni2–O23 174.33(7)°. 

The C–O bond distances of the five-membered chelate ring amounting to 1.260(3) and 1.257(3) Å for O11–C11 and O21–C21, respectively, assume the keto form of the ligand. These distances are significantly shorter than C11–O11 and C21–O21 of 1.296(4) and 1.289(4) Å in **1**∙2MeOH∙MeCN. This is additionally sustained by the crystallographically proven existence of the intermolecular hydrogen bonds via the –NH group of the central hydrazone linkage, =N–NH–(C=O)–. The –OH group of the first ligand (labelled as O14) is deprotonated, proving the electroneutrality of the complex molecule. This is confirmed by the closeness of the possible hydrogen position of that –OH group with the neighbouring hydrogen atoms positions: H14…H24 of approximately 1.2 Å. 

**Figure 4:**
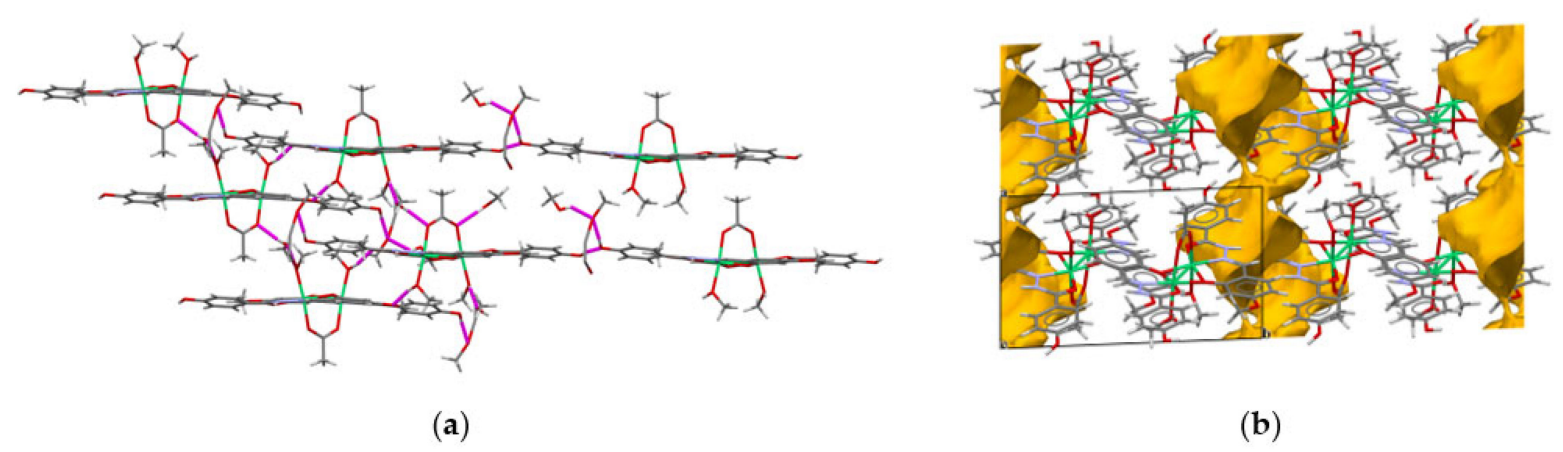
(**a**) View of supramolecular structure of **2∙**4.7MeOH. The hydrogen bonds (dashed magenta lines) net complex molecules and methanol molecules of crystallisation via the O–H**···**O-type bonds. (**b**) The alternating supramolecular architecture of solvent and complex molecules spreading along the *b* axis (yellow voids represent channels formed by solvent molecules). Color code: Ni-green, N-blue, O-red, C-gray, H-white.

The crystal structure of **2**∙4.7MeOH reveals a rich variety of hydrogen bonds (Figure 4), which are established both between the complex molecules and the solvent ones (Figure S1). This leads to the formation of a complex network of O–H···O-type hydrogen bonds (being in the O···O distance range of 2.559(2)–2.797(4) Å) and the N–H···O ones, which are additionally supported by the weaker C–H···O (range 3.206(3)–3.696(5) Å) and C–H···N (C4ME–H4MB···N12 of 3.376(3) Å) interactions. A close inspection of supramolecular architecture reveals that solvent molecules are accommodated within channels, spreading parallel to the *c* axis and alternating with complex molecules in an AB manner (Figure 4b). The AB linkage based on hydrogen bonds occurs along the *b* axis.

#### 2.1.3. Structure of [Ni_4_(HL^H^)_2_(L^H^)_2_(OAc)_2_]∙4MeOH·0.63H_2_O·0.5CH_3_CN·HOAc

The tetranuclear core of the cubane topology of **3***∙*4MeOH·0.63H_2_O·0.5MeCN·HOAc (Figure 3b) is built up of four nickel centres and four phenoxido oxygen atoms situated at the apexes of a non-symmetrical cube in a manner of two penetrated Ni4 and O4 tetrahedra. In that way, the phenoxido oxygen atoms of six-membered chelate rings span three nickel centres. The cubane-like topology results in Ni1···Ni2 and Ni3···Ni4 separations of 2.957(1) Å and 2.960(1) Å, respectively. Each nickel atom is coordinated octahedrally by a NO5 donor atoms set derived from four *ONO* tridentate hydrazone ligands and two bridging acetate ions, each of them spanning two nickel centres. 

Two ligands exist in the hydrazonato L^2−^ form and two of them in hydrazidato HL^−^ form. This is evidenced by the existence of a hydrogen position at the non-coordinating nitrogen atom and participation in hydrogen bonding formation (N42–H4N···O5ME and N32–H3N···O1W hydrogen bonds of 2.848(4) and 2.803(6)Å, respectively, and by C–O bond distances of five-membered chelate rings, which are shorter for the HL^−^ (O31–C31 1.249(1) and O41–C41 1.251(1) Å) than for the L^2−^ form (O11–C11 1.275(1) and O21–C21 1.265(1) Å, Tables S2 and S4). The shortest nickel-to-donor atom bond distance is of the Ni–N type, while all Ni–O bond distances are longer than 2.0 Å, with the Ni–O(phenoxido) being the longest ones (varying in the range of 2.041 (1)–2.172 (1) Å, Table S4).

The three solvent methanol molecules are situated in general positions, while the atoms of two methanol molecules are refined with the 0.5 occupancy factor. The unit cell also contains one half acetonitrile molecule per complex unit, which is not included in hydrogen bond formation. The oxygen atom of one of the methanol molecules is disordered over two positions within the unit cell. Accordingly, the water molecule O1W is refined with the 0.63 occupancy factor (see Experimental Section). 

**Figure 5:**
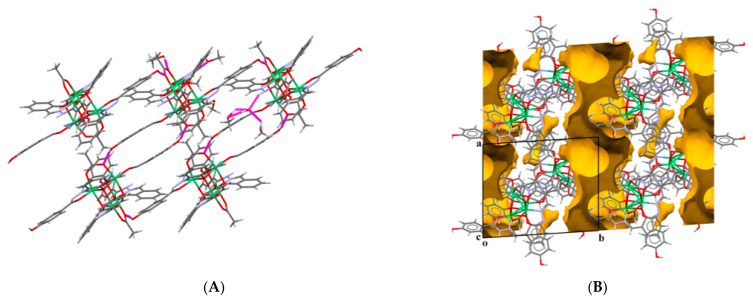
(**A**) Crystal packing view of **3**∙4MeOH∙0.63H_2_O∙0.5MeCN∙HOAc showing O–H···O and N–H···O intermolecular hydrogen bonds by magenta dashed lines. (**B**) The existence of two types of voids (denoted in yellow) is obvious; one in the form of channels that accommodates non-disordered solvent molecules and the other that are discrete, accommodating disordered solvent molecules. Color code: Ni-green, N-blue, O-red, C-gray, H-white.

The presence of acetic acid instead of the acetate ion is concluded based on C–O bond geometry (1.34 and 1.29 Å) as well as clear and reasonable geometry of the HL^−^ and L^2−^ ligands form within the Ni_4_L_4_ core, which compensates the electroneutrality of the complex molecule. Moreover, the –OH group of the acid participates in hydrogen bond formation (O6–H6···O2ME of 2.721(14) Å; Table S2). The crystal structure is dominated, except the two abovementioned N–H···O bonds, by plenty of O–H···O (Figure 5A) and C–H···O-type hydrogen bonds (for a detailed description, see the Supplementary Materials, Figure S2, Table S2). 

The crystal structure of **3***∙*4MeOH·0.63H_2_O·0.5MeCN·HOAc reveals two types of supramolecular channels along the *a* axis (Figure 5B). One type of channel accommodates solvent molecules whose atoms are not disordered but hydrogen-bonded with complex molecules, and the other one is fulfilled with the disordered O1ME methanol molecule, which is not linked by hydrogen bonds with complex molecules (see text above and Table S2). It seems that non-hydrogen bond formation with the O1ME methanol molecule enables its spatial flexibility over two positions within channels.

### 2.2. Mononuclear Nickel(II) Complexes 

The synthesis conditions were further modified by performing the reaction of Ni(OAc)_2_∙4H_2_O and the corresponding hydrazone ligand either with the addition of pyridine (instead of Et_3_N) or without the addition of base. Good-quality red crystals of the mononuclear complexes [Ni(L^3OMe^)(py)] (**4**), [Ni(L^4OMe^)(py)] (**5**), and [Ni(L^H^)(py)] (**6**) were obtained under solvothermal conditions upon addition of pyridine to the reaction mixture in methanol. Otherwise, under reflux conditions, powdered solids deposited. A systematic variation in the pyridine amount did not affect the product or the number of coordinated pyridine ligands. Additionally, in pyridine, clusters **1**–**3** transform into the mononuclear complexes **4**–**6**, respectively. Again, one hydrazone ligand is bound to Ni(II) in the tridentate fashion, and the remaining coordination sites are occupied by one pyridine molecule.

Reported structurally characterised nickel(II) complexes with a tridentate hydrazone ligand and pyridine are either in a distorted octahedral or a square planar environment ([Ni(L)(py)_3_] [48,49] or [Ni(L)(py)] [50,51], respectively. To investigate the possibility of the existence of octahedral complexes with three pyridine molecules, quantum chemical calculations of those theoretically built complexes were performed and standard Gibbs energies of binding were compared to the standard Gibbs energies of binding calculated for experimentally obtained square planar complexes [52]. In each case, the difference is negative (Table 1), meaning that square planar complexes with one pyridine molecule are more stable than corresponding octahedral complexes with three pyridine molecules.

Synthesis of the complexes [Ni(HL^3OMe^)_2_] (**7**), [Ni(HL^4OMe^)_2_] (**8**), and [Ni(HL^H^)_2_] (**9**) was carried out in methanol by using Ni(OAc)_2_∙4H_2_O and the corresponding aroylhydrazone in a 1:2 ratio. Effort was made to crystallise the complexes in various solvents. Unfortunately, only powdered compounds precipitated in all cases. The magnetic susceptibilities of the stable complexes **7–9** were measured out by Evans’s balance. Effective magnetic moments were *μ*_eff_ = 2.93 μ_B_, 2.98 μ_B_, and 3.04 μ_B_ at 29,215 K, respectively. The observed paramagnetism is consistent with the octahedral Ni^II^ coordination environment in complexes. We, also, applied a different synthetic approach. Synthesis by the metal exchange procedure starting from [MoO_2_(L)(EtOH)] and Ni(OAc)_2_∙4H_2_O produced the crystalline sample of [Ni(HL^H^)_2_]∙2MeOH (**9**∙2MeOH). Complex [Ni(HL^3OMe^)_2_]∙MeOH (**7**∙MeOH) was also obtained as a solvate by this procedure, but despite many attempts, good-quality crystals suitable for single-crystal X-ray diffraction experiments were not obtained. Each time, the crystals isolated were extremely thin green plates that lose solvent molecules at room temperature. Solvent removal in **9**∙2MeOH and **7**∙MeOH resulted in the destruction of the original crystalline order resulting in either a product of decreased crystallinity or an amorphous material. The resulting materials obtained upon desolvation of **9**∙2MeOH and **7**∙MeOH gave the same IR spectra as **9** and **7**, respectively, obtained upon the reaction of Ni(OAc)_2_ and the corresponding ligand. 

#### Structures of [Ni(L^3OMe^)(py)], [Ni(L^4OMe^)(py)], and [Ni(HL^H^)_2_]∙2MeOH

**Figure 6:**
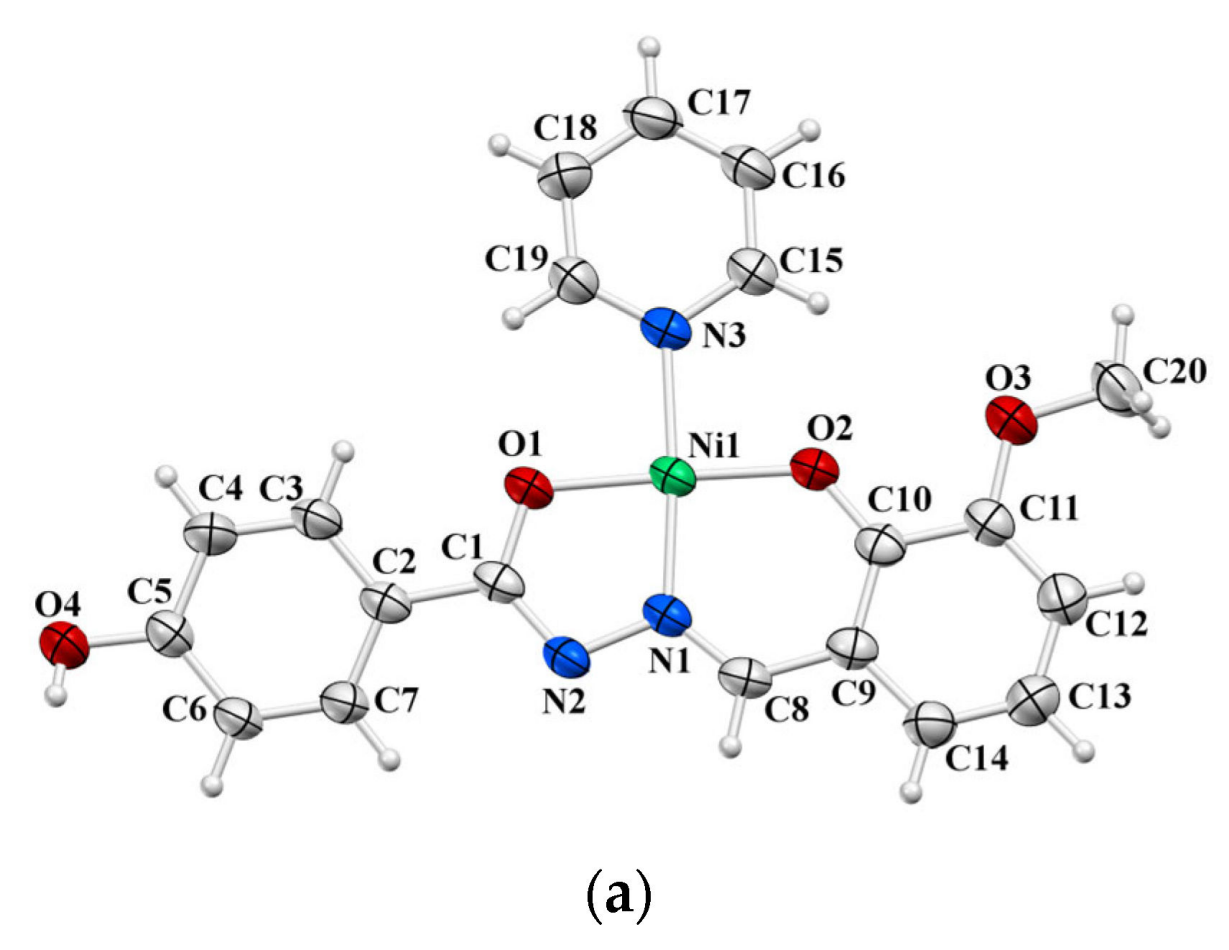

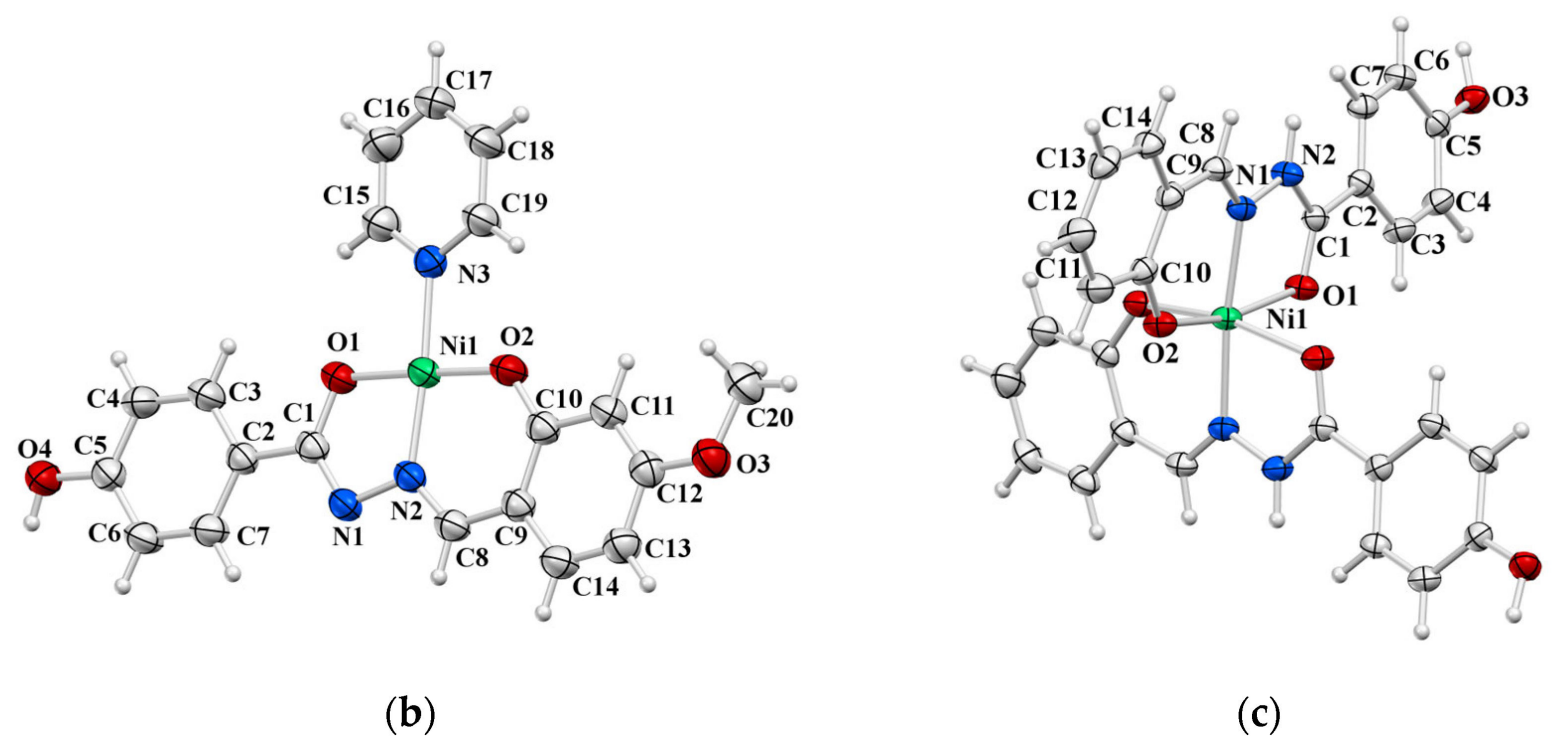
ORTEP view of molecular structures of: (**a**) **4**, (**b**) **5**, and (**c**) **9**∙2MeOH. The displacement ellipsoids are drawn at the 50% probability level at 296(2) K for **4** and **5**, and 150(2) for K **9**∙2MeOH. The MeOH solvent molecules are omitted for clarity.

In **4** and **5**, the Ni(II) ion is coordinated by hydrazonato ligand L^2−^ in an tridentate manner and by a pyridine nitrogen atom, thus forming a square-planar coordination (Figure 6a and Figure 6b, respectively). The coordination sphere of the Ni1 atom in **9**·2MeOH is an octahedral one formed by two tridentate ligands related by a crystallographically imposed inversion centre at the Ni1 atom (Figure 6c). Nickel to donor atoms bond distances are significantly longer, as expected, for octahedral (2.011(2) Å–2.0983(18) Å) than for square-planar mononuclear nickel complexes (1.815(1) Å–1.945(2) Å) (see Table S5). 

While the ligands in **4** and **5** complexes exist in the hydrazonato =N–N=(C–O)– L^2–^ form (O1–C1 1.296(2) and 1.299(2) in **4** and **5**), the electroneutrality of complex **9**·2MeOH along with bond distances within the central part of the ligand (C1=O1 1.252(3) Å, C1–N2 1.353(3) Å, and N1–N2 1.389(3) Å) suggest the hydrazidato =N–NH–(C=O)– HL^−^ ligand form. The N2–H2···O4 intermolecular hydrogen bond of 2.808(3) Å with the non-coordinating N2 atom acting as the proton donor confirms additionally the HL^−^ ligand form in complex **9**·2MeOH (see text below).

The molecules of complexes **4** and **5** are joined via O4–H4O···N2 intermolecular hydrogen bonds into zig-zag chains spreading along the *b* axis. The other H-bonds are of the C–H···O or C–H···N type, but all of them are formed with the N2 and O4 atoms as proton acceptors (Figures S3 and S4, Table S2). The complex molecules in **9**·2MeOH are mutually linked via O3–H3O···O2 intermolecular hydrogen bonds into 2D chains of hydrogen-bonded rings spreading along the *b* axis (Figures S5 and S6). One methanol molecule is attached to a complex molecule via an N2–H2···O4 intermolecular hydrogen bond and with another methanol molecule via an O4–H4O···O5 intermolecular hydrogen bond with the O4 methanol oxygen atom simultaneously acting as a proton donor and proton acceptor.

### 2.3. Hybrid Organic–Inorganic Compound Based on Polyoxomolybdate

The reaction of [MoO_2_(L^4OMe^)(MeOH)] with Ni(OAc)_2_∙4H_2_O in methanol proceeds differently, and the hybrid organic–inorganic compound based on polyoxomolybdate [Ni_2_(HL^4OMe^)_2_(CH_3_OH)_4_][Mo_4_O_10_(OCH_3_)_6_] (**10**) was obtained without isolation of the corresponding mononuclear complex. The crystals lose coordinated solvent molecules at room temperature resulting in the formation of [Ni_2_(HL^4OMe^)_2_][Mo_4_O_10_(OCH_3_)_6_]. The hybrid salt exhibits an interesting vapour-induced colour change between green and red-brown on the absorption/desorption of methanol molecules. Despite numerous attempts, we could not produce the analogous hybrids using H_2_L^H^ and H_2_L^3OMe^ ligands. Hybrid **10** based on polyoxomolybdate represents a rare example of a mixed bridged dinuclear cluster.

#### Structure of [Ni_2_(HL^4OMe^)_2_(CH_3_OH)_4_][Mo_4_O_10_(OCH_3_)_6_]

**Figure 7:**
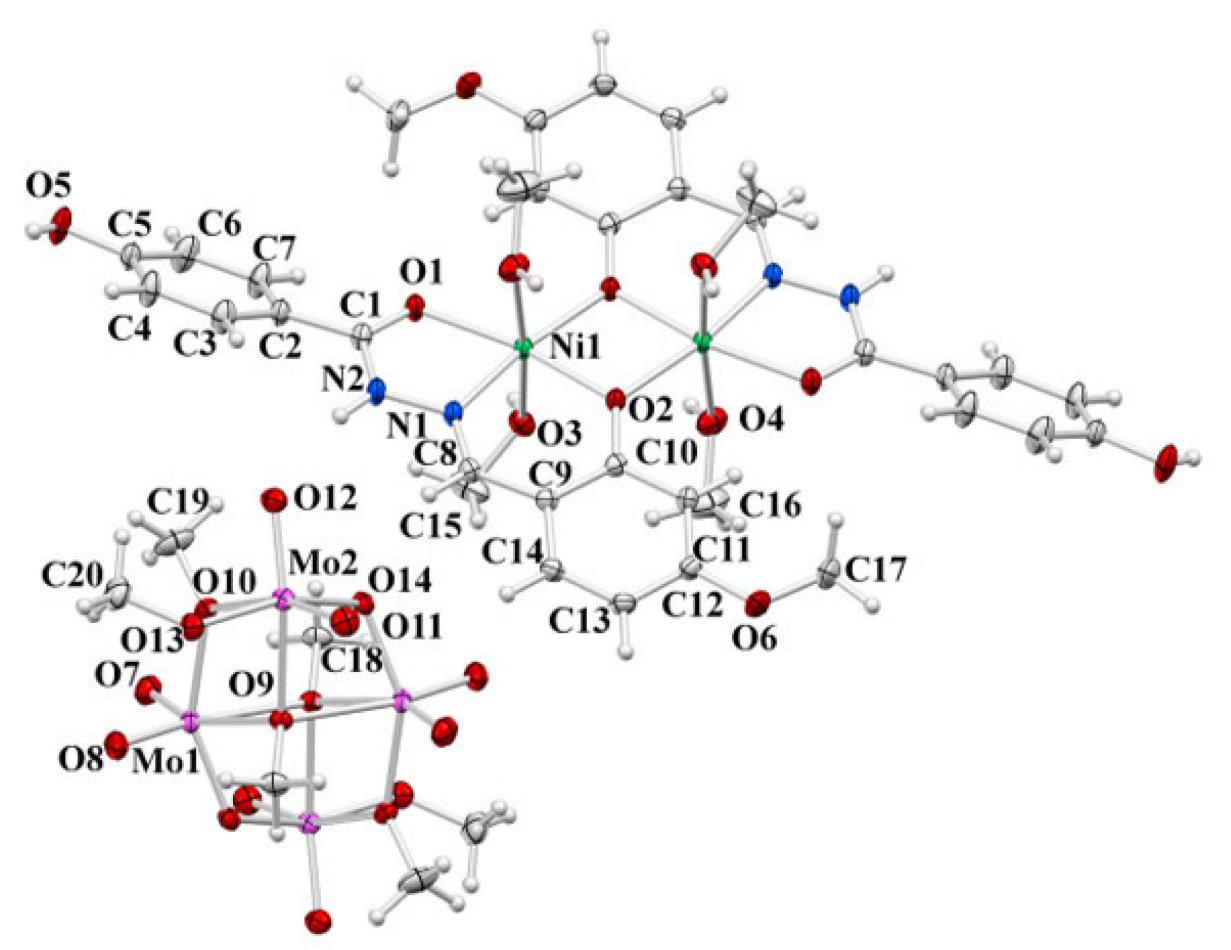
ORTEP view of molecular structure of **10**. The displacement ellipsoids are drawn at the 50% probability level at 150(2) K. The hydrogen atoms are drawn as spheres of arbitrary radius. The atom-numbering crystallographic scheme has been applied.

The asymmetric unit of the hybrid compound **10** consists of [Ni(L^4OMe^)(CH_3_OH)_2_] and [Mo_2_O_5_(OCH_3_)_3_] moieties (Figure 7). The complex cation formed by the crystallographically imposed inversion centre is built up of dinuclear nickel units (Ni…Ni separation of 3.092(1) Å) with each nickel centre octahedrally surrounded by NO5 donor atoms from one monoanionic tridentate *ONO* hydrazidato HL^−^ ligand form, two coordinated methanol molecules, and the phenoxido oxygen atom from another hydrazone ligand. Bond distances are as expected for octahedral nickel dinuclear bridging complexes and are found to be similar to these in complex **2∙**4.7MeOH. The Ni–O(CH_3_OH) bond distances with methanol molecules are among the longest ones in the coordination sphere (Table S6). The bond distances within the five-membered chelate ring indicate the existence of the ligand in the hydrazidato tautomeric =N–NH–(C=O)– form along with the existence of hydrogen at the N2 nitrogen atom implying a monoanionic ligand form. The clear confirmation of the predominant keto form of the C1–O1 bond is a distance of 1.251(3) Å. The complex anion is of the [Mo_4_O_10_(OCH_3_)_6_]^2−^ type of known geometry.

**Figure 8:**
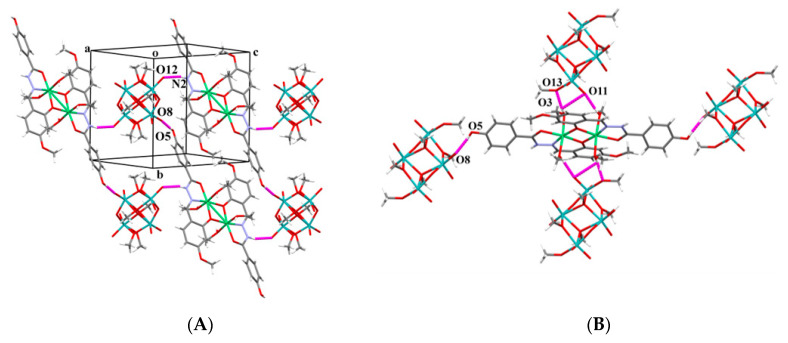
(**A**) Supramolecular linkage of cations and anions into 1D zig-zag chain parallel to *c* axis via N2-H2N···O12 and via O5-H5O···O8 intermolecular hydrogen bond along *a* axis into 1D chains; (**B**) trapped [Ni_2_(HL^4OMe^)_2_(CH_3_OH)_4_]^2+^ cation by four [Mo_4_O_10_(OCH_3_)_6_]^2−^ anions.

The cations and anions are linked into a 1D zig-zag chain via the N2–H2N···O12 intermolecular hydrogen bond formed between the –NH ligand part and the terminal oxo O12 atom of the anion (Table S2; Figure 8A). The chains spread along the *c* axis. Another H-bond O5–H5O···O8 formed between the hydroxyl O5 group of the ligand and the terminal O8 atom of the anion joins cations and anions along the *a* axis into 1D chains. 

Two other bonds join [Mo_4_O_10_(OCH_3_)_6_]^2−^ and [Ni_2_(HL^4OMe^)_2_(CH_3_OH)_4_]^2+^ moieties: O3–H3O···O13 hydrogen bond (Figure 8B) defined between the coordinated methanol molecule and the methoxy group of the anion and O4–H4O···O11 between another coordinated methanol molecule and the terminal O11 oxygen atom of the anion (Figure S2; Table S2). Other hydrogen bonds are of the C–H···O type and longer than 3.2 Å. Thus, the complex cation [Ni_2_(L^4OMe^)_2_(CH_3_OH)_4_]^2+^ has been trapped by hydrogen bonds with four [Mo_4_O_10_(OCH_3_)_6_]^2−^ anions (Figure 8B).

### 2.4. Thermal Analysis

Crystals of **1**∙2MeOH∙MeCN, **2**∙4.7MeOH, and **3**∙MeOH∙0.63H_2_O∙0.5MeCN∙HOAc are unstable and easily lose coordinated and uncoordinated solvent molecules even at −15 °C. This can be associated with their position within the channels of their crystal structures. However, at room temperature, further destruction of the structure occurred. The mass loss corresponding to the first step in the TG curves of the residual [Ni_3_(L^3OMe^)_2_(OAc)_2_], [Ni_2_(HL^4OMe^)(L^4OMe^)(OAc)_2_], and [Ni_4_(HL^H^)_2_(L^H^)_2_(OAc)_2_] was related to the decomposition of the coordinated acetato ligand. This process occurred in the range of 25–111 °C, 25–116 °C, and 25–131 °C, respectively. On further heating, the decomposition of the hydrazone ligand started at 262 °C for **1**, 208 °C for **2**, and 204 °C for **3**. The final residue was identified as NiO. 

Compounds [Ni(L)(py)] (**4**–**6**) with coordinated pyridine showed considerable thermal stability. Furthermore, grinding of samples does not change the crystal structure as seen from PXRD patterns (Figure S7). Release of a pyridine molecule in **4** and **5** upon heating was accompanied by complex decomposition (in the range of 248–365 °C and 264–369 °C, respectively). The first step in the thermogravimetric curve of **6** was related to the loss of pyridine (243–291 °C), followed by a significant weight loss (397–401 °C) due to ligand decomposition.

The unsolvated products **7**–**9** were stable in a certain temperature range when they started to decompose (PXRD patterns of **7**–**9** are given in Figure S8). During the decomposition of the complexes [Ni(HL^3OMe^)_2_] (**7**), [Ni(HL^4OMe^)_2_] (**8**), and [Ni(HL^H^)_2_] (**9**), mass losses occurred in the range of 274–344 °C, 324–397°C, and 319–413°C, respectively. However, the solvated compounds [Ni(HL)_2_]∙*x*MeOH lost their solvent molecules already upon standing at room temperature. A complete solvent loss in **9**∙2MeOH occurred even in a freezer at −15 °C. The first step in the thermogravimetric curve of **7**∙MeOH (25–84 °C) was related to the loss of the MeOH molecule. 

In the case of [Ni_2_(HL^4OMe^)_2_(CH_3_OH)_4_][Mo_4_O_10_(OCH_3_)_6_] (**10**), the cation decomposition was accompanied by the anion decomposition without a stable intermediate product. The corresponding thermograms obtained for all compounds are given in Figures S9–S18.

### 2.5. Spectroscopic Characterisation

The spectra of complexes **7–10** showed a set of bands related to a singly deprotonated ligand, while those of **1**, **4**–**6** displayed bands due to a doubly deprotonated ligand. However, the spectra of **2** and **3** showed two sets of bands belonging to singly and doubly deprotonated ligands [43]. IR spectra of the oligonuclear and mononuclear compounds are given in Figures S19–S22.

The band characteristic for the C=O group at, ca., 1645 cm^−1^ (seen in the IR spectrum of H_2_L) shifted to 1557–1540 cm^−1^ in the spectra of the complexes, suggesting coordination of the hydrazidato ligand through the carbonyl oxygen atom [43,53]. On the other hand, the presence of a new band in the range of 1297–1273 cm^−1^, due to stretching vibrations of the C–O bond, suggested tautomerism (=N–NH–(C=O)– ⇆ =N–N=(C–OH)–), deprotonation, and coordination of hydrazonato form through the oxygen atom. 

In the IR spectra of the ligands, vibration bands belonging to C=N_imine_ and C–O_phenolic_ groups were found at, ca., 1630 cm^−1^ and 1355 cm^−1^, respectively. In the case of the HL^−^ ligand, these bands shifted to 1615–1604 cm^−1^ and 1379–1328 cm^−1^. respectively. For L^2–^ ligand, they were found at lower wave numbers (in the range 1610–1598 cm^−1^ and 1285–1236 cm^−1^, respectively). This finding indicates the coordination of the ligands to the metal centre through the nitrogen and oxygen atoms of these two groups.

Complexes **1**–**3** showed strong stretching frequencies in the 1561–1556 cm^−1^ and 1443–1331 cm^−1^ regions due to the attribution to the *ν*_asym_(OCO) and *ν*_sym_(OCO) vibrations, respectively [54,55]. The presence of [Mo_4_O_10_(OCH_3_)_6_]^2−^ in **10** was identified by the stretching frequencies characteristic for the *cis*-MoO_2_^2+^ structural unit (found at 912 cm^−1^ and 892 cm^−1^) [56,57]. The compound was also identified by the appearance of bands at ~710 cm^−1^ assigned to *δ*(O_b_–Mo = O_t_) of the tetramolybdate anion.

## 3. Materials and Methods

### 3.1. Preparative Part 

Ligands H_2_L^H^, H_2_L^3OMe^∙H_2_O, and H_2_L^4OMe^∙H_2_O were prepared by the reaction of 4-hydroxybenzhydrazide with an appropriate aldehyde under mechanochemical conditions [40]. NMR spectral data are given in Table S7 together with the NMR numbering scheme (Scheme S3). Complexes [MoO_2_(L)(EtOH)] were obtained according to the procedure described in the literature [58]. Commercially available solvents and chemicals purchased from Aldrich (Amsterdam, Netherlands) were used without further purification. 

#### 3.1.1. Synthesis of [Ni_3_(L^3OMe^)_2_(OAc)_2_(MeOH)_2_]∙2MeOH∙MeCN (1∙2MeOH∙MeCN)

A solution of Ni(OAc)_2_∙4H_2_O (0.1 g, 0.40 mmol) in 10 mL of a 1:1 (*v*/*v*) mixture of methanol and acetonitrile was added to a solution of H_2_L^3OMe^∙H_2_O (0.08 g, 0.27 mmol) in 5 mL of the methanol and acetonitrile mixture (1:1, *v/v*). Afterward, 25 μL of triethylamine was added to the reaction mixture. The solution was heated at 50–60 °C for one hour and the resulting bright green solution was allowed to stand at room temperature for a few days. Green crystals of **1** were filtered and dried in a desiccator (at −15 °C) up to the constant weight. They easily lost methanol and acetonitrile molecules and were analysed as [Ni_3_(HL^3OMe^)_2_(OAc)_2_]. Yield: 0.09 g; 70%. Anal. Calcd. for C_34_H_30_N_4_Ni_3_O_12_ (862.701): C, 47.36 H, 3.51; N, 6.49%. Found: C, 47.1; H, 3.35; N, 6.12%. TG: calc. for NiO 25.97%, found 25.50%. Selected IR data (cm^−1^): 1610 (C=N), 1595 (ring C=C), 1274 (C–O), 1237 (C–O_phenoxido_), 1560, 1431 (COO).

#### 3.1.2. Synthesis of [Ni_2_(HL^4OMe^)(L^4OMe^)(OAc)(MeOH)_2_]∙4.7MeOH (2∙4.7MeOH)

The complex was prepared in the same manner as **1** using Ni(OAc)_2_∙4H_2_O (0.1 g, 0.40 mmol) and H_2_L^4OMe^∙H_2_O (0.12 g, 0.4 mmol). Green crystals of **2** were filtered and dried in a desiccator (at −15 °C) up to the constant weight. They lost solvent molecules easily and were analysed as [Ni_2_(HL^4OMe^)(L^4OMe^)(OAc)_2_]. Yield: 0.08 g; 53%. Anal. Calcd. for C_32_H_28_N_4_Ni_2_O_10_ (745.972): C, 51.52 H, 3.78; N, 7.51%. Found: C, 51.2; H, 3.35; N, 7.25%. TG: calc. for NiO 20.05%, found 19.69%. Selected IR data (cm^−1^): 1613 (C=N), 1598 (ring C=C), 1540 (C=O), 1382 (C–N), 1379 (C–O_phenoxido_), 1556, 1441 (COO).

#### 3.1.3. Synthesis of [Ni_4_(HL^H^)_2_(L^H^)_2_(OAc)_2_]∙4MeOH∙0.63H_2_O∙0.5MeCN∙HOAc (3∙4MeOH∙0.63H_2_O∙0.5MeCN∙HOAc) 

The complex was prepared in the same manner as **1** using Ni(OAc)_2_∙4H_2_O (0.1 g, 0.40 mmol) and H_2_L^H^ (0.10 g, 0.4 mmol). Green crystals of **3** were filtered and dried in a desiccator (at −15 °C) up to the constant weight. They lost solvent molecules easily and were analysed as [Ni_4_(HL^H^)(L^H^)(OAc)_2_]. Yield: 0.1 g; 73%. Anal. Calcd. for C_60_H_48_N_8_Ni_4_O_16_ (1371.841): C, 52.53 H, 3.53; N, 8.17. Found: C, 52.3; H, 3.25; N, 7.9%. TG: calc. for NiO 21.78%, found 22.05%. Selected IR data (cm^−1^): 1612 (C=N)_HL_, 1598 (C=N)_L_, 1598 (ring C=C), 1540 (C=O)_HL_, 1378 (C–N)_HL_, 1365 (C–O_phenoxido_)_HL_, 1296 (C–O)_L_, 1276 (C–O_phenoxido_)_L_, 1561, 1443 (COO).

#### 3.1.4. Synthesis of [Ni(L^3OMe^)(py)] (**4**)

Ni(OAc)_2_∙4H_2_O (0.1 g, 0.40 mmol), H_2_L^3OMe^∙H_2_O (0.12 g, 0.40 mmol), and 75 μL of py were suspended in 20 mL of a 1:1 (*v/v*) mixture of methanol and acetonitrile in a 35 mL Teflon liner, which was sealed in an autoclave and heated at 110 °C for 2 h. The solution was allowed to cool slowly, resulting in the formation of a dark red crystalline product. The obtained product was filtered and dried up to constant weight. Yield: 0.14 g; 83%. Anal. Calcd. for C_20_H_17_N_3_NiO_4_ (422.06): C, 56.91; H, 4.06; N, 9.96%. Found: C, 56.71; H, 3.85; N, 9.71%. TG: calc. for NiO 17.76%, found 17.9%. Selected IR data (cm^−1^): 1612 (C=N)_py_, 1602 (C=N), 1598 (ring C=C), 1273 (C–O), 1245 (C–O_phenoxido_). 

#### 3.1.5. Synthesis of [Ni(L^4OMe^)(py)] (**5**)

The complex was prepared in the same manner as **4** using Ni(OAc)_2_∙4H_2_O (0.1 g, 0.40 mmol), H_2_L^4OMe^∙H_2_O (0.12 g, 0.40 mmol), and 75 μL of py. Yield: 0.13 g; 77%. Anal. Calcd. for C_20_H_17_N_3_NiO_4_ (422.06): C, 56.91; H, 4.06; N, 9.96%. Found: C, 56.75; H, 3.81; N, 9.62%. TG: calc. for NiO 17.76%, found 17.5%. Selected IR data (cm^−1^): 1614 (C=N)_py_, 1608 (C=N), 1598 (ring C=C), 1274 (C–O), 1253 (C–O_phenoxido_). 

#### 3.1.6. Synthesis of [Ni(L^H^)(py)] (**6**)

The complex was prepared in the same manner as **4** using Ni(OAc)_2_∙4H_2_O (0.1 g, 0.40 mmol), H_2_L^H^ (0.10 g, 0.40 mmol), and 75 μL of py. Yield: 0.1 g; 63%. Anal. Calcd. for C_19_H_15_N_3_NiO_3_ (392.034): C, 58.21; H, 3.86; N, 10.72%. Found: C, 58.01; H, 3.63; N, 10.64%. TG: calc. for py 20.18%, found 19.95%; calc. for NiO 19.12%, found 19.3%. Selected IR data (cm^−1^): 1611 (C=N)_py_, 1601 (C=N), 1593 (ring C=C), 1276 (C–O), 1247 (C–O_phenoxido_). 

#### 3.1.7. Synthesis of [Ni(HL^3OMe^)_2_] (**7**)

*Method A*: A mixture of Ni(OAc)_2_∙4H_2_O (0.1 g, 0.40 mmol) and H_2_L^3OMe^∙H_2_O (0.24 g, 0.80 mmol) in methanol (35 mL) was refluxed for one hour. The obtained precipitate of **7** was filtered, rinsed with methanol, and dried in a desiccator. Yield: 0.21 g; 83%.

*Method B*: A solution of [MoO_2_(L^3OMe^)(EtOH)] (0.055 g, 0.12 mmol) in 50 mL of methanol was added to a solution of Ni(OAc)_2_∙4H_2_O (0.03 g, 0.12 mmol) in 5 mL of methanol. The mixture was allowed to stand at room temperature for a few days. Green crystals of **7**∙MeOH were filtered and dried in a desiccator. Yield: 0.008 g; 21%. Crystals easily lost solvent molecules at room temperature and were analysed as [Ni(HL^3OMe^)_2_]. 

Calcd. for C_30_H_26_N_4_NiO_8_ (629.242): C, 57.26; H, 4.16; N, 8.90%. Found: C, 57.05; H, 3.82; N, 8.59%. For TG analysis, crystals of **7**∙MeOH were transferred on a dry filter paper into a desiccator and then placed in a freezer (at −15 °C). TG: calc. for MeOH 4.85%, found 4.39%; NiO 11.30%, found 11.42%. Selected IR data (cm^−1^): 1615 (C=N), 1599 (ring C=C), 1554 (C=O), 1386 (C−N), 1367 (C–O_phenoxido_). 

#### 3.1.8. Synthesis of [Ni(HL^4OMe^)_2_] (**8**)

A mixture of Ni(OAc)_2_∙4H_2_O (0.1 g, 0.40 mmol) and the aroylhydrazone H_2_L^4OMe^∙H_2_O (0.24 g, 0.80 mmol) in dry methanol (35 mL) was refluxed for one hour. The obtained precipitate was filtered, rinsed with methanol, and dried in a desiccator. Yield: 0.20, 79%. Anal. Calcd. for C_30_H_26_N_4_NiO_8_ (629.242): C, 57.26; H, 4.16; N, 8.90%. Found: C, 57.0; H, 3.90; N, 8.71%. TG: calc. for NiO 11.87%, found 12.1%. Selected IR data (cm^−1^): 1606 (C=N), 1594 (ring C=C), 1556 (C=O), 1378 (C–N), 1328 (C–O_phenoxido_). 

#### 3.1.9. Synthesis of [Ni(HL^H^)_2_] (**9**)

*Method A*: Complex **9** was prepared in the same manner as **7** using Ni(OAc)_2_∙4H_2_O (0.1 g, 0.40 mmo), H_2_L^H^ (0.20 g, 0.80 mmol). Yield: 0.18 g; 79%. 

*Method B*: Complex **9**∙2MeOH was prepared in the same manner as **7**∙MeOH using [MoO_2_(L^H^)(EtOH)] (0.051 g, 0.12 mmol) and Ni(OAc)_2_∙4H_2_O (0.03 g, 0.12 mmol). Yield: 0.01 g; 3%. Green crystals easily lost solvent molecules at room temperature and were analysed as [Ni(HL^H^)_2_]. 

Anal. Calcd. for C_28_H_22_N_4_NiO_6_ (569.191): C, 59.08; H, 3.90; N, 9.84%. Found: C, 58.83; H, 3.65; N, 10.12%. TG: calc. for NiO 13.00%, found 13.35%. Selected IR data (cm^−1^): 1604 (C=N), 1597 (ring C=C), 1543 (C=O), 1382 (C–N), 1333 (C–O_phenoxido_). 

#### 3.1.10. Synthesis of [Ni_2_(HL^4OMe^)_2_(CH_3_OH)_4_][Mo_4_O_10_(OCH_3_)_6_] (**10**)

The cluster was prepared in the same manner as **7**∙MeOH using [MoO_2_(L^4OMe^)(EtOH)] (0.055 g, 0.12 mmol) and Ni(OAc)_2_∙4H_2_O (0.03 g, 0.12 mmol). Green crystals of **10** were filtered and dried in a desiccator (at −15 °C) up to the constant weight and analysed as [Ni_2_(HL^4OMe^)_2_][Mo_4_O_10_(OCH_3_)_6_]. Yield: 0.024 g; 56%. Anal. Calcd. for C_36_H_44_Mo_4_N_4_Ni_2_O_24_ (1417.893): C, 30.49; H, 3.13; N, 3.95%. Found: C, 30.19; H, 3.25; N, 3.65%. TG: calc. for NiO and MoO_3_ 10.55% and 40.66%, respectively; found: 50.69% total residual mass of oxides. Selected IR data (cm^−1^): 1610 (C=N), 1596 (ring C=C), 1557 (C=O), 1285 (C–O_phenoxido_), 912, 892 (MoO_2_), 732, 713, 702 (Mo–O_b_). 

### 3.2. Physical Methods 

#### 3.2.1. X-ray Crystallography—Powder Diffraction 

The powder X-ray diffraction (PXRD) data were collected using a Malvern Panalytical Aeris powder diffractometer (Malvern Panalytical B.V., Almelo, The Netherlands) in the Bragg–Brentano geometry with a PIXcel^1D^ detector, using Cu*K_α_* radiation (*λ* = 1.5406 Å). Samples were contained on a Si sample holder. Powder patterns were collected at room temperature in the range of 5–40° (2*θ*). The data were collected and visualised utilising the Malvern Panalytical HighScore Software Suite [59]. PXRD patterns of the stable mononuclear compounds are given in Figures S7 and S8.

#### 3.2.2. Single-Crystal X-ray Diffraction Experiments 

Selected crystallographic and refinement data for structures **1**∙2MeOH∙MeCN, **2**∙4.7MeOH, **3**∙4MeOH∙0.63H_2_O∙0.5MeCN∙HOAc, **4**, **5**, **9**∙2MeOH, and **10**, obtained by the single-crystal X-ray diffraction experiments, are reported in Table S1. The data for all structures were collected by ω-scans on an Oxford Xcalibur diffractometer (Oxford Diffraction Ltd., Abingdon, UK) equipped with a 4-circle kappa geometry goniometer, CCD Sapphire 3 detector, and graphite-monochromated MoK_α_ radiation (*λ* = 0.71073 Å) at various temperatures: 150(2) K for **1**∙2MeOH∙MeCN, **2**∙4.7MeOH, **3**∙4MeOH∙0.63H_2_O∙0.5MeCN∙HOAc, **9**∙2MeOH, and **10** structures and at 296(2) K for **4** and **5**.

The data reduction was performed using the CrysAlisPro software package [60]. Structure solutions were accomplished by using direct methods followed by differential Fourier syntheses. Structural refinement was performed on *F*^2^ by the weighted full-matrix least-squares method. Non-hydrogen atoms were refined anisotropically. Programs SHELXT-2014 [61] and SHELXL-2014 [62] integrated into the WinGX [63] software system were used to solve and refine the structures. 

All hydrogen atoms attached to the carbon atoms were fixed in calculated positions as riding on the corresponding carbon atoms with the temperature-dependent C–H bond distances, while the others (–OH or –NH) were found as peaks of small electron densities in difference electron-density Fourier maps and refined with the restrained bond distances and assigned isotropic displacement parameters being 1.2 times larger than the equivalent isotropic displacement parameters of the parent atoms. Complex **2** contained 4.7 molecules of methanol crystallisation molecules per complex molecule. The 0.7 methanol molecule was accomplished by occupancy refinement as a free variable for atoms O1ME and C1ME. The O5M atom of one of the methanol molecules was disordered over two positions A and B, whose occupancies were refined in the ratio 0.7:0.3, which was determined due to the presence of the 0.7MeOH molecule and the short distance between the water oxygen atom and the minor position of the methanol oxygen atom, i.e., both the water and the methanol oxygen atom minor positions alternate in the 0.7:0.3 ratio within the unit cell. The hydrogen atom at O5MB was not found. 

Complex **3**∙4MeOH∙0.63H_2_O∙0.5MeCN∙HOAc contained five methanol molecules of crystallisation, with three of them situated in general positions and two of them refined with the atom’s occupancy factor of 0.5, with half an acetonitrile molecule per complex molecule and 0.63 water molecules and one acetate acid molecule situated at general positions. The O1ME atom of one of the methanol molecules was disordered over two positions, and occupancies were refined in the ratio 63:37. In accordance with the disorder of the O1Me atom, the water molecule O1W atom occupancy was set to 0.63. The O1ME and O1W hydrogen atoms were not found in difference Fourier maps in the final stages of refinement. The geometrical calculations and structural analyses were performed using PARST [64] and MERCURY [65] programs. Drawings were made by MERCURY. Main geometrical features (selected bond distances and angles) along with hydrogen bond geometry for the structures are given in Tables S2–S7.

#### 3.2.3. Thermal, Spectroscopic, and Magnetic Measurements 

Thermogravimetric (TG) analysis was carried out with a Mettler-Toledo TGA/SDTA851e thermobalance (Mettler Toledo, Columbus, OH, USA) using aluminium crucibles. All experiments were recorded in a dynamic atmosphere with a flow rate of 200 cm^3^ min^−1^. Heating rates of 5 K min^−1^ were used for all investigations. Elemental analyses were provided by the Analytical Services Laboratory of the Ruđer Bošković Institute, Zagreb. 

Fourier Transform Infrared spectra (FT-IR) were recorded in KBr pellets with a Perkin-Elmer Spectrum Two spectrophotometer (Waltham, MA, USA). Spectra were recorded in the spectral range between 4500 and 450 cm^−1^. NMR spectra of hydrazones were recorded on a Bruker Avance III HD 400 spectrometer operating at 400 MHz equipped with a broadband observed (BBO) Prodigy cryoprobe and z-gradient accessory. Compounds were dissolved in DMSO-d_6_ and measured in 5 mm NMR tubes at 298 K with TMS as an internal standard. The sample concentration was 10 mg/mL (Table S8, Scheme S3). 

The magnetic measurements were carried out by a Sherwood Scientific magnetic susceptibility balance.

#### 3.2.4. Quantum Chemical Calculation 

Calculations of geometry optimisation and harmonic vibrational frequencies were carried out using the Gaussian 16 program package [66]. Geometry optimisation for ground states were performed using hybrid functionals B3LYP [67,68] with the D3 version of Grimme’s dispersion [69] in combination with the 6-31G(d) basis set. For all optimised structures, harmonic frequencies were calculated to ensure that obtained geometries correspond to the minimum on the potential energy surface. The standard Gibbs energies were calculated at *T* = 298.15 K and *p* = 101325 Pa. In all nickel(II) complexes, nickel was in the low-spin (singlet) state.

## 4. Conclusions

Hydrazone is successfully used as *ONO* chelating and *O*-bridging ligands in the synthesis of the nickel(II) complexes. The appropriate synthetic approach, i.e., stoichiometric ratio of Ni(II) salt and H_2_L, usage of Et_3_N, an appropriate reaction temperature, and other conditions, enables formation of the acetato- and phenoxido-bridged trinuclear [Ni_3_(L^3OMe^)_2_(OAc)_2_(MeOH)_2_]∙2MeOH∙MeCN (**1**∙2MeOH∙MeCN), dinuclear [Ni_2_(HL^4OMe^)(L^4OMe^)(OAc)(MeOH)_2_]∙4.7MeOH (**2**∙4.7MeOH), and tetranuclear [Ni_4_(HL^H^)_2_(L^H^)_2_(OAc)_2_]∙4MeOH·0.63H_2_O·0.5MeCN·HOAc (**3**∙4MeOH·0.63H_2_O·0.5MeCN ·HOAc) clusters. Bridging carboxylate ligands are required for construction of the clusters whereas the hydrazone ligand structure plays an important role in maintaining the specific structure. A different nuclearity of complexes is simultaneously followed by the outcome of the tautomeric form (hydrazonato or hydrazidato) and extent of the ligand deprotonation. As seen from X-ray diffraction and TG analysis, the solvent molecules are located in channels from which they can easily escape.

Absence of the bridging ligands results in the formation of the mononuclear complexes [Ni(HL)_2_] (**7**–**9**). When pyridine is used, the ligands coordinate only one nickel atom and the square-planar geometry is favoured as found for complexes [Ni(L)(py)] (**4**–**6**). Quantum chemical calculations reveal the stability of square planar complexes in comparison to the corresponding octahedral complexes. In each case, the standard Gibbs energies of binding are lower for square planar than for octahedral complex in a range of −70 to −80 kJ mol^−1^. The mononuclear complexes **7**∙MeOH, **9**∙2MeOH, and hybrid organic–inorganic compound [Ni_2_(HL^4OMe^)_2_(CH_3_OH)_4_][Mo_4_O_10_(OCH_3_)_6_] (**10**) are achieved by the metal-exchange procedure starting from [MoO_2_(L)(EtOH)].

## Figures and Tables

**Table 1:** Calculated differences of standard Gibbs energies of binding ΔΔ_b_*G*° between square planar [Ni(L)(py)] and octahedral ([Ni(L)(py)_3_] complexes.

Complex	ΔΔ_b_*G*°
4	−76.46
5	−72.37
6	−81.75

## Data Availability

Crystallographic datasets for the structures **1**∙2MeOH∙MeCN, **2**∙4.7MeOH, **3**∙4MeOH∙0.63H_2_O∙0.5MeCN∙HOAc, **4**, **5**, **9**∙2MeOH, and **10** are available through the Cambridge Structural Database with deposition numbers CCDC 1573444-1573450. These data can be obtained free of charge via https://www.ccdc.cam.ac.uk/conts/retrieving.html (accessed on 15 January 2023) (or from the CCDC, 12 Union Road, Cambridge CB2 1EZ, UK; Fax: +44 1223 336033; E-mail: deposit@ccdc.cam.ac.uk).

## Supplementary Materials

The following supporting information can be downloaded at: https://www.mdpi.com/article/10.3390/ijms24031909/s1.
