# Supplementary material for: Ligand-Modulated Nuclearity and Geometry in Nickel(II) Hydrazone Complexes: From Mononuclear Complexes to Acetato- and/or Phenoxido-Bridged Clusters
Source: Int J Mol Sci. 2023 Jan 18;24(3):1909. doi: 10.3390/ijms24031909 (PMC9915932; doi:10.3390/ijms24031909)
Supplement: Supplementary file 1 [file ijms-24-01909-s001.zip › ijms-2161873-supplementary.pdf]

Supporting Materials

Ligand-Modulated Nuclearity and Geometry in Nickel(II) Hydrazone Complexes: From Mononuclear Complexes to Acetato and/or Phenoxido Bridged Clusters

Višnja Vrdoljak, Tomica Hrenar, Mirta Rubčić, Gordana Pavlović, Tomislav Friganović and Marina Cindrić

Contents

Scheme 2

Scheme S1 Hydrazidato and hydrazonato ligand forms and reversible deprotonation.
Scheme S2 Schematic reperesntation of oligonuclear clusters and mononuclear complexes.

X-Ray Crystallography. Single crystal diffraction. 4

Hydrogen-bonded network in **2**·4.7MeOH
Hydrogen-bonded network in **3**·4MeOH·0.63H_2O·0.5MeCN·HOAc
Figure S1. Complex hydrogen-bonded network observed in **2**·4.7MeOH
Figure S2 Crystal packing in **3**·4MeOH·0.63H_2O·0.5MeCN·HOAc
Figure S3 View of zig-zag chain spreading along b axis of complex molecules **4**.
Figure S4 View of zig-zag chain spreading along b axis of complex molecules **5**.
Figure S5 View of crystal structure of **9**·2MeOH showing 2D chains of hydrogen bonded rings.
Figure S6 Crystal packing in **9**·2MeOH.
Table S1 Crystallographic data and structure refinement data.
Table S2 Selected bond lengths [Å] and angles [°] for complex **1**·2MeOH·MeCN.
Table S3 Hydrogen bonds and interactions geometry (Å)
Table S4 Selected bond lengths [Å] and angles [°] for complex **2**·4.7MeOH
Table S5 Selected bond lengths [Å] and angles [°] for complex **3**·4MeOH·0.63H_2O·0.5MeCN·HOAc
Table S6 Selected bond lengths [Å] and angles [°] for complex **4**, **5** and **9**·2MeOH
Table S7 Selected bond lengths [Å] and angles [°] for complex **10**

Powder X-ray diffraction 25

Figure S7. PXRD patterns of the mononuclear nickel(II) complexes [Ni(L)(py)] (**4-6**)
Figure S8. PXRD patterns of mononuclear nickel(II) complexes [Ni$(HL)_2$] (**7-9**)

Thermal analysis 26

Figure S9 TGA thermogram of desolvated trinuclear nickel(II) cluster [$Ni_3(L^{3OMe})_2(OAc)_2$].
Figure S10 TGA thermogram of desolvated dinuclear nickel(II) cluster [$Ni_2(HL^{4OMe})(L^{4OMe})(OAc)_2$].
Figure S11 TGA thermogram of desolvated tetranuclear nickel(II) cluster [$Ni_4(HL^H)_2(L^H)_2(OAc)_2$].
Figure S12 TGA thermogram of mononuclear nickel(II) complex [$Ni(L^{3OMe})(py)$] (**4**).
Figure S13 TGA thermogram of mononuclear nickel(II) complex [$Ni(L^{4OMe})(py)$] (**5**).
Figure S14 TGA thermogram of mononuclear nickel(II) complex [$Ni(L^H)(py)$] (**6**).
Figure S15 TGA thermogram of mononuclear nickel(II) complex [$Ni(HL^{3OMe})_2$]·MeOH (**7**·MeOH).
Figure S16 TGA thermogram of desolvated mononuclear nickel(II) complex [$Ni(HL^{4OMe})_2$] (**8**).
Figure S17 TGA thermogram of desolvated mononuclear nickel(II) complex [$Ni(HL^H)_2$] (**9**).
Figure S18 IR spectra of desolvated hybrid based on polyoxomolybdate [$Ni_2(HL^{4OMe})_2$] [$Mo_4O_{10}(OCH_3)_6$].

IR spectroscopy 31

Figure S19 IR spectra of acetato and phenoxido bridged di-, tri- and tetranuclear nickel(II) clusters: [$Ni_3(L^{3OMe})_2(OAc)_2$], [$Ni_2(HL^{4OMe})(L^{4OMe})(OAc)_2$], and [$Ni_4(HL^H)_2(L^H)_2(OAc)_2$]
Figure S20 IR spectra of mononuclear nickel(II) complexes **4-6**.
Figure S21 IR spectra of mononuclear nickel(II) complexes **7-9**
Figure S22 IR spectrum of hybrid organic-inorganic compound based on polyoxomolybdate.

Ligands 35

Table S8 ^{1}H and ^{13}C chemical shifts (ppm) of compounds $\mathbf{H_2L^H}$, $\mathbf{H_2L^{3OMe}}$·H_2O and $\mathbf{H_2L^{4OMe}}$·H_2O.
Scheme S3 The structural formula of H_2L with the NMR numbering scheme.

Scheme

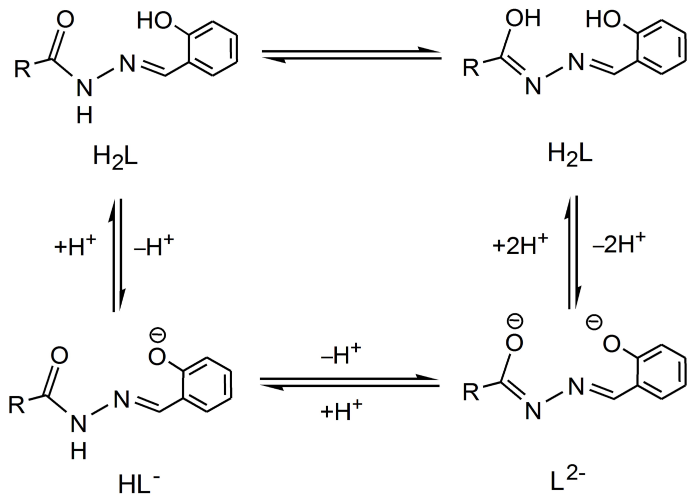

Scheme S1 Hydrazidato =N−NH−(C=O)− and hydrazonato =N−N=(C−O)− ligand forms and reversible deprotonation.

(a)

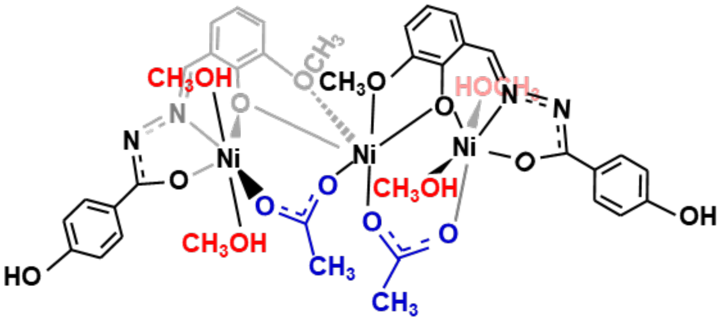

(b)

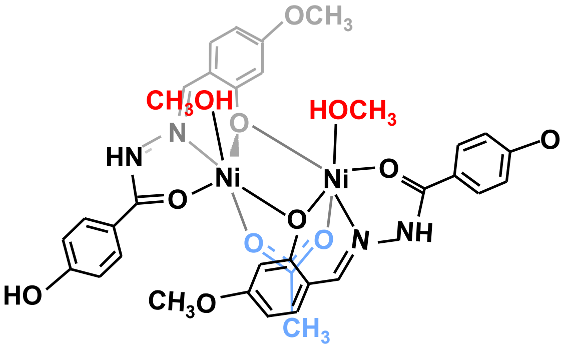

(c)

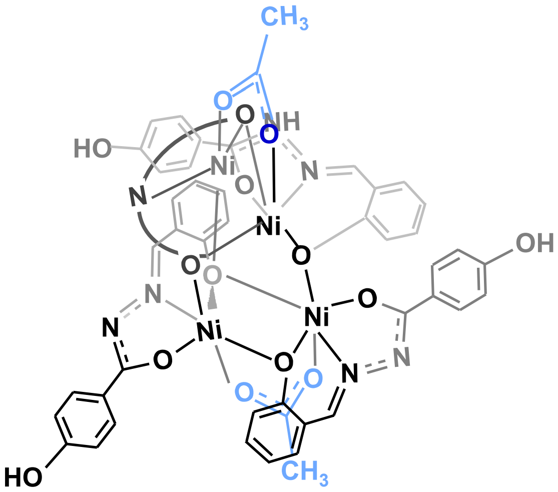

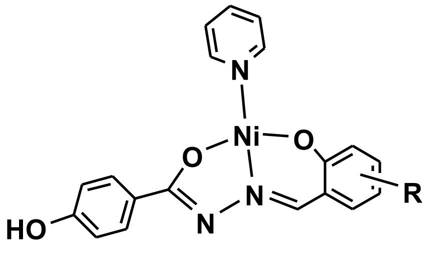

(d)

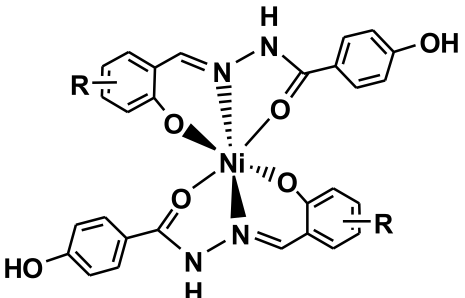

(e)

Scheme S2 Schematic representation of nickel(II) oligonuclear acetato and phenoxido bridged clusters: (a) [$Ni_3(L^{3OMe})_2(OAc)_2(MeOH)_2$] (**1**), (b) [$Ni_2(HL^{4OMe})(L^{4OMe})(OAc)(MeOH)_2$] (**2**), and (c) [$Ni_4(HL^{H})_2(L^{H})_2(OAc)_2$] (**3**) (in tetranuclear cluster **3**, for the sake of clarity, one ligand is represented schematically by a curved line with *ONO* donor atoms), and mononuclear complexes: (d) [$Ni(L^{R})(py)$] (**4-6**) and (e) [$Ni(HL^{R})_2$] (**6-9**), (R = OCH_3 or H).

X-Ray Crystallography. Single crystal diffraction.

Hydrogen-bonded network in 2·4.7MeOH

The complex molecules are linked *via* intermolecular hydrogen bonds formed with two methanol solvent molecules both acting with their oxygen atoms as proton donors and proton acceptors (Fig. 4, Fig S1). There are two hydrogen bonds which involve –NH ligand group as proton donor and the methanol oxygen atoms O2ME and O3ME: N12−H12N···O2ME and N22−H22N···O3ME (Table S2). Simultaneously, these methanol oxygen atoms form two O−H···O type of intermolecular hydrogen bond: one O3ME−H3ME···O2 with the acetate oxygen atom O2 and another with the methanol oxygen atom O1ME: O2ME−H2ME···O1ME (Table S2).

The third methanol molecule is clasped to one complex molecule *via* O4ME−H4ME···O1 hydrogen bond, while the O4ME oxygen atom is simultaneously engaged as proton acceptor in O23−H23O···O4ME hydrogen bond with the oxygen atom O23 of coordinated methanol molecule of another complex molecule (Table S2). These later hydrogen bonds spread along *c* axis, while the former are situated along *b* axis.

The other coordinated methanol molecule form O13−H13O···O25 intermolecular hydrogen bond with the methoxy O25 atom of another complex molecule (Table S2). Both hydroxyl groups of ligand, deprotonated O14 and protonated O24, participate in hydrogen bond. The O24−H24O···O14 hydrogen bond between two hydroxyl groups joins supramolecular aggregate of two hydrogen bonded complex molecules into infinite chains along *c* axis (Table S2).

In such a way the complex molecules along with the solvent methanol molecules are supramolecularly networked by O−H···O type of hydrogen bonds (being in the acceptable O···O distance range of 2.559(2) - 2.797(4) Å), supported by the weaker C−H···O (range 3.206(3) - 3.696(5) Å) and C−H···N (C4ME–H4MB···N12 of 3.376(3) Å) hydrogen bonds.

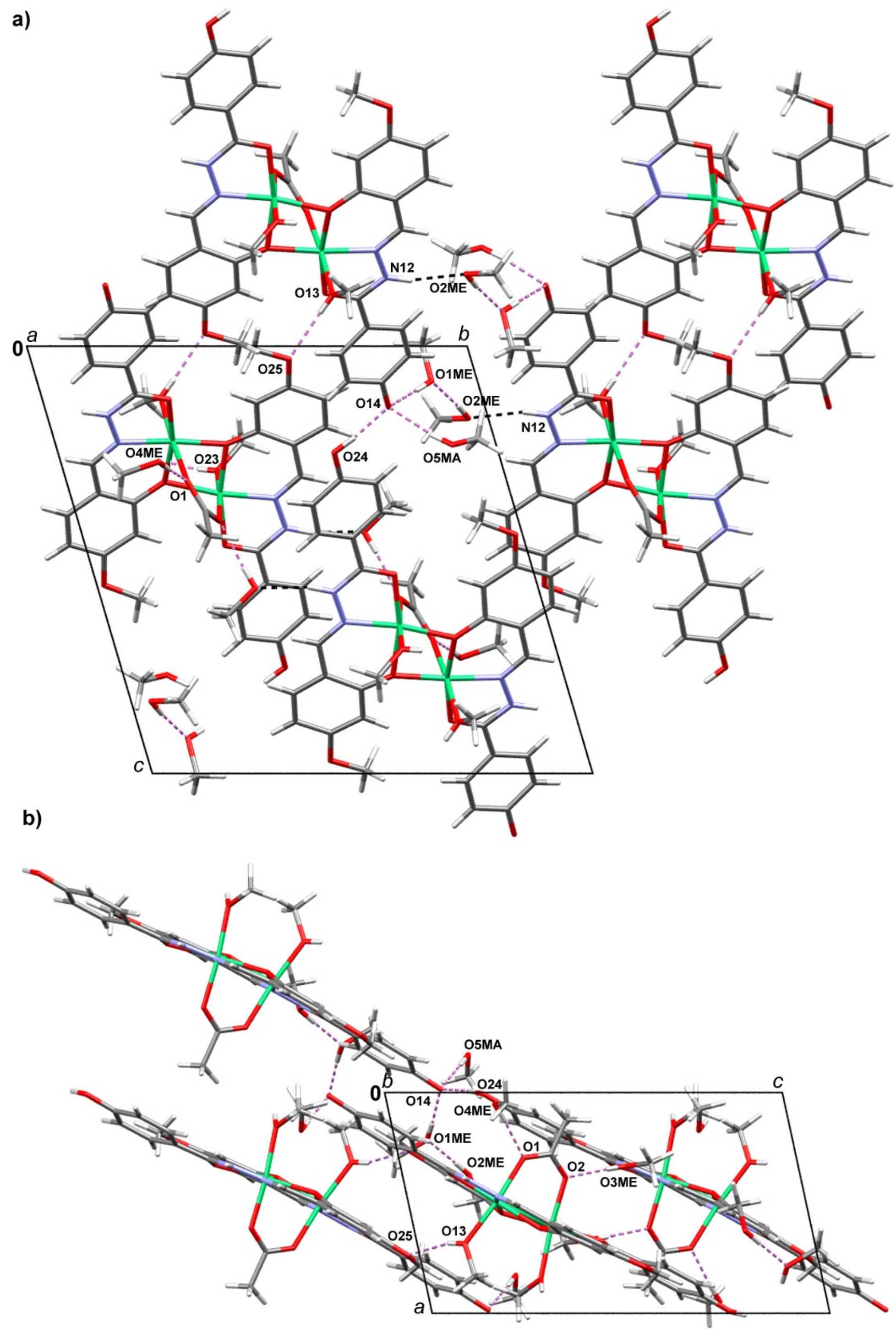

Figure S1. Complex hydrogen-bonded network observed in **2**·4.7MeOH: a) view down the *a*-axis; b) view down the *b*-axis. Hydrogen bonds of the O–H···O type are shown as purple dashed lines, whereas the N–H···O hydrogen bonds are highlighted by black dashed lines. Color scheme: O red, N blue, Ni green, C dark gray, H light gray.

Hydrogen-bonded network in 3·4MeOH·0.63H$_2$O·0.5MeCN·HOAc

The O−H···O hydrogen bonds between two complex molecules (such as O13−H13O···O4, O23−H23O···O1) are realized between coordinated acetate oxygen atoms and the hydroxyl groups of ligand, while the other are formed with solvent molecules (O43−H43O···O4ME, O5ME−H5ME···O3) or the acetate acid oxygen atoms (O33−H33O···O5). The solvent and acid molecules are linked mutually by hydrogen bonds, too (O6−H6···O2ME, O2ME−H2ME···O1W). The geometry of O−H···O type of intermolecular hydrogen bonds are in the range 2.559(5) - 2.764(6) Å. The other hydrogen bonds are of the C−H···O type being in the range 3.117(5) (C211−H026···O11) - 3.492(6) Å (C2−H2A···O23).

a)

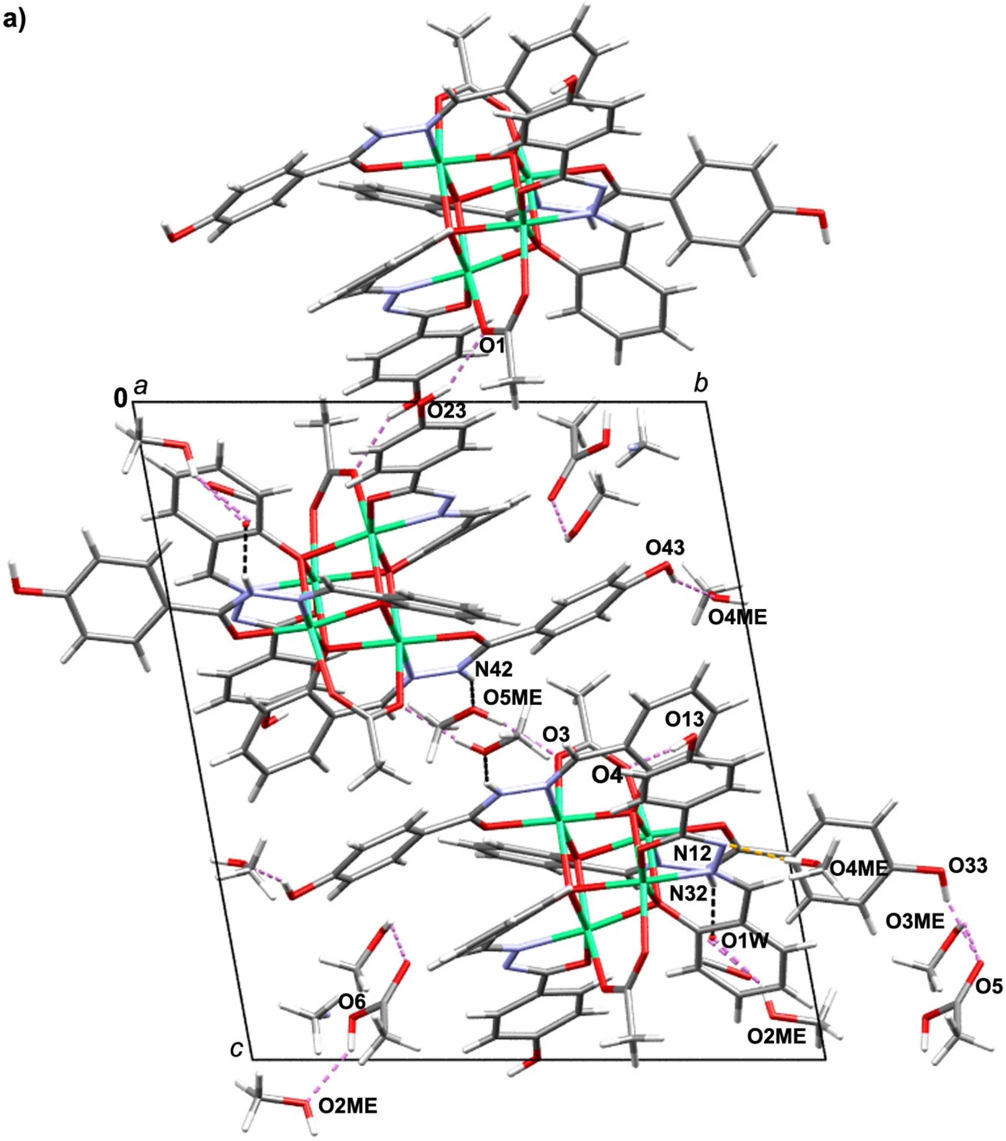

b)

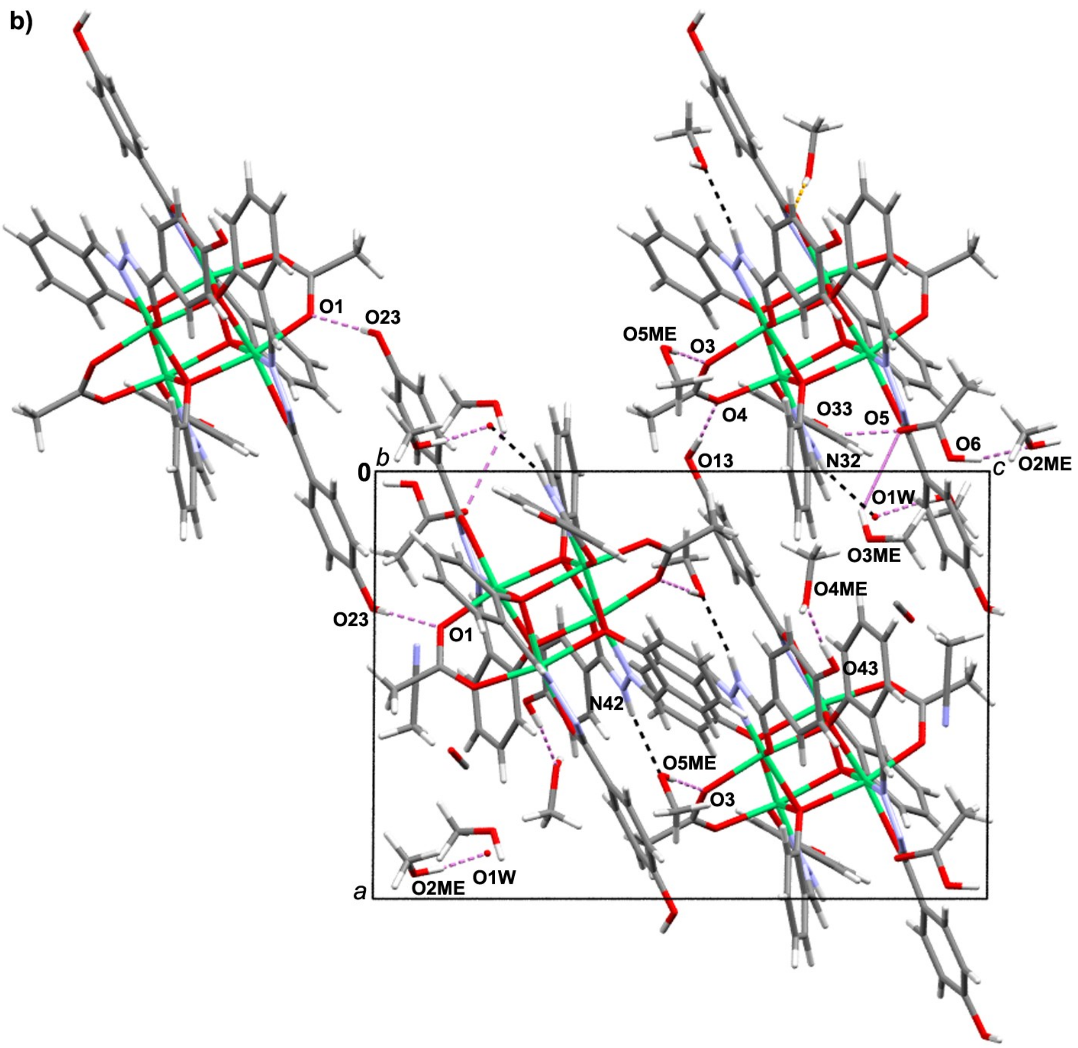

Figure S2 Crystal packing in **3**·4MeOH·0.63H_2O·0.5MeCN·HOAc viewed down the: a) *a*-axis, and b) *b*-axis. Hydrogen bonds of the O–H···O type are shown as purple dashed lines, the N–H···O as black dashed lines, while the O–H···N ones are highlighted by orange dashed lines. Color scheme: O red, N blue, Ni green, C dark gray, H light gray.

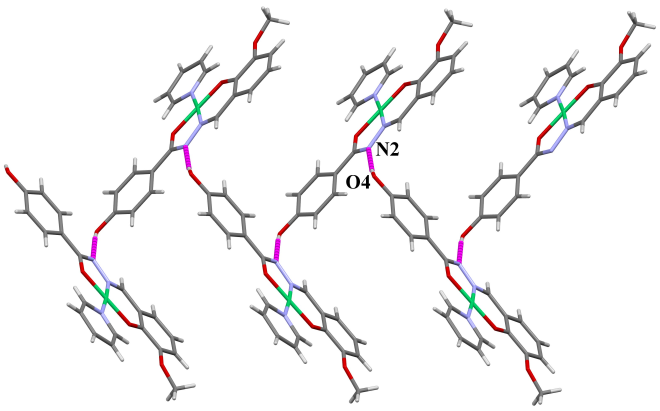

Figure S3 View of *zig-zag* chain spreading along *b*-axis of complex molecules **4** *via* O4−H4O···N2 intermolecular hydrogen bonds (dashed magenta lines)

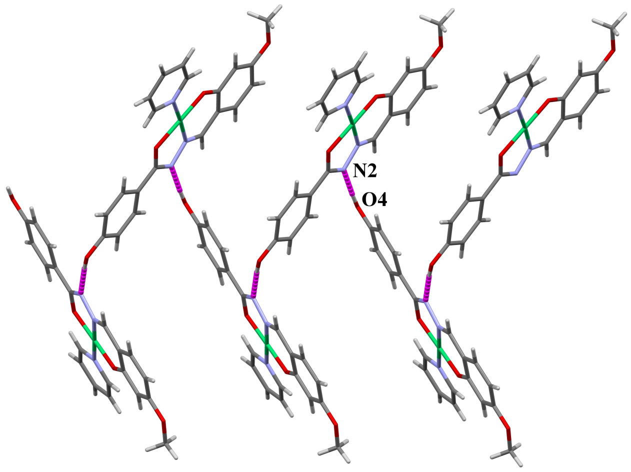

Figure S4 View of *zig-zag* chain spreading along *b*-axis of complex molecules **5** *via* O4−H4O···N2 intermolecular hydrogen bonds (dashed magenta lines)

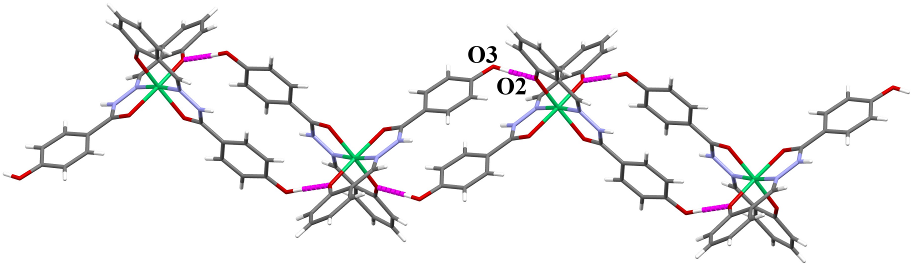

Figure S5 View of crystal structure of **9**·2MeOH showing 2D chains of hydrogen bonded rings spreading along *b*-axis.

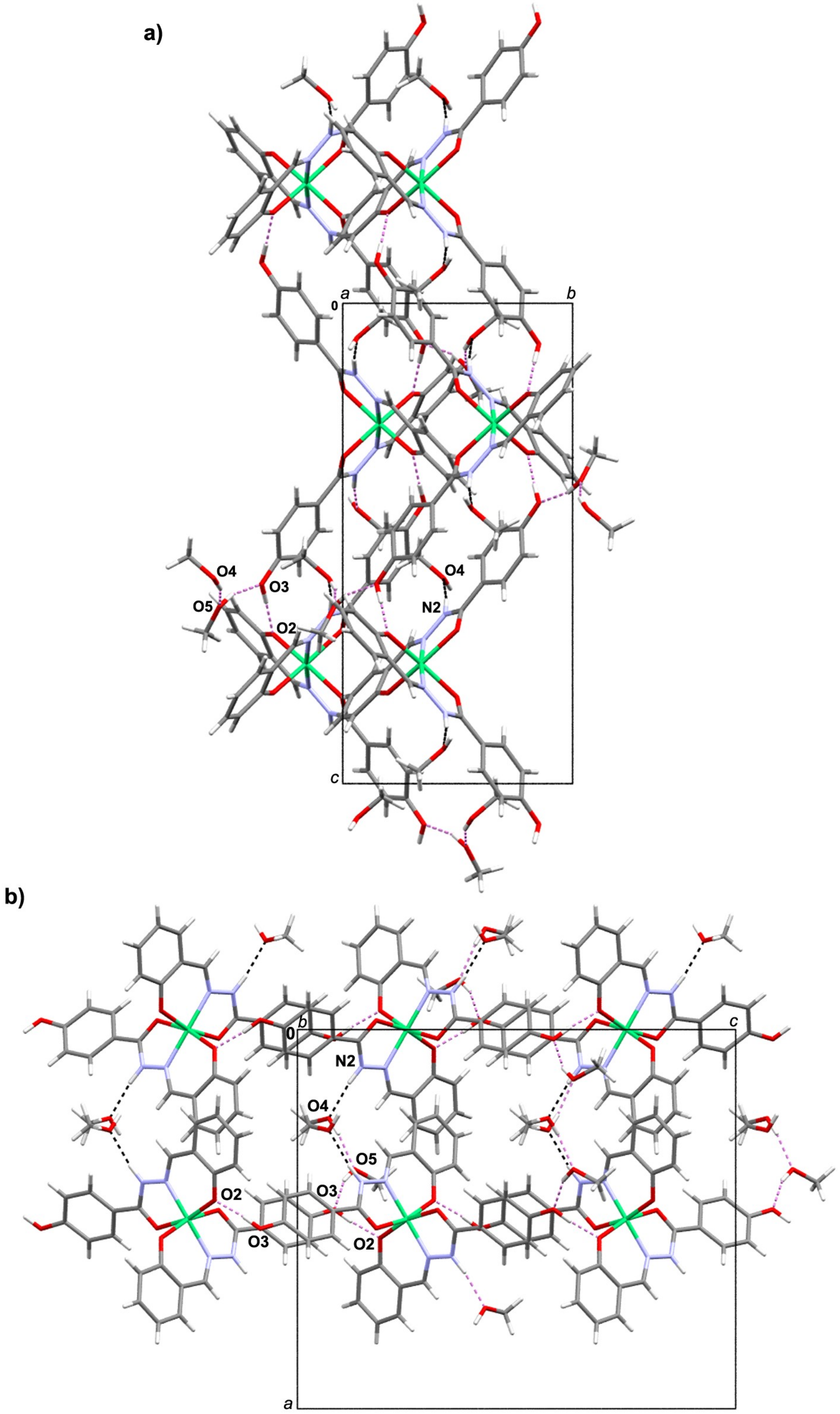

Figure S6 Crystal packing in **9**·2MeOH: a) view down the *a*-axis; b) view down the *b*-axis. Supramolecular 2D chains formed through O3–H3O···O2 interaction are mutually joined *via* N2–H2···O4 and O4–H4O···O5 hydrogen bonds, which involve (crystallization) methanol molecules. Hydrogen bonds of the O–H···O type are shown as purple dashed lines and the N–H···O as black dashed lines. Color scheme: O red, N blue, Ni green, C dark gray, H light gray.

Table S1 Crystallographic data for compounds **1**·2MeOH·MeCN, **2**·4.7MeOH, **3**·4MeOH·0.63H_2O·0.5MeCN·HOAc, **4**, **5**, **9**·2MeOH and **10**.

**Complex**	**1**·2MeOH·MeCN	**2**·4.7MeOH	**3**·4MeOH·0.63H_2O·0.5MeCN ·HOAc	**4**	**5**	**9**·2MeOH	**10**
Chemical formula	$C_{42}H_{57}N_5Ni_3O_{18}$	$C_{77.40}H_{109}N_8Ni_4O_{33.40}$	$C_{134}H_{131}N_{17}Ni_8O_{45.26}$	$C_{20}H_{17}N_3NiO_4$	$C_{20}H_{17}N_3NiO_4$	$C_{32}H_{38}N_4NiO_{10}$	$C_{40}H_{60}Mo_4N_4Ni_2O_{28}$
M_r	1096.05		3173.39	422.07	422.07	697.37	1546.10
Crystal system, habit and colour	Monoclinic, green, plate	Triclinic, green, prism	Triclinic, green, plate	Monoclinic, green, plate	Monoclinic, green, prism	Orthorhombic, green, plate	Triclinic, green, needle
Crystal dimensions / mm^3	0.25 × 0.38 × 0.03	0.18 × 0.25 × 0.40	0.25 × 0.32 × 0.05	0.15 × 0.14 × 0.43	0.40 × 0.35 × 0.33	0.32 × 0.30 × 0.17	0.31 × 0.11 × 0.09
Space group	$P\,2_1/n$	$P\,\bar{1}$	$P\,\bar{1}$	$P\,2_1/n$	$P\,2_1/n$	$P\,b\,c\,n$	$P\,\bar{1}$
Z	4	1	1	4	4	4	1
Unit cell parameters:							
a /Å	9.6868 (3)	8.9675 (2)	12.5439 (3)	13.5603 (8)	13.3279 (3)	17.3202 (6)	10.1912 (4)
b /Å	23.5870 (7)	15.8211 (4)	15.7644 (4)	9.2622 (5)	8.3575 (2)	9.5808 (4)	11.7410 (4)
c /Å	21.8848 (5)	16.3976 (4)	18.2789 (4)	14.6706 (9)	16.3635 (4)	20.0329 (8)	12.1450 (4)
α/°	90	72.751 (2)	79.640 (2)	90	90	90	99.528 (3)
β/°	95.030 (2)	76.644 (2)	89.696 (2)	99.732 (6)	94.925 (2)	90	96.565 (3)
γ/°	90	83.944 (2)	86.395 (2)	90	90	90	102.602 (3)
V /$Å^3$	4981.0 (2)	2159.99 (9)	3548.57 (15)	1816.08 (19)	1815.97 (7)	3324.3 (2)	1381.44 (9)
D_{calc} /g cm^{-3}	1.462	1.477	1.485	1.544	1.544	1.393	1.858
μ /mm^{-1}	1.20	0.95	1.13	1.10	1.10	0.65	1.64
F(000)	2288	1009	1640	872	872	1464	776
Index range	$h = -12 \to 8$ $k = -31 \to 31$ $l = -26 \to 28$	$h = -11 \to 11$ $k = -20 \to 17$ $l = -17 \to 20$	$h = -17 \to 17$ $k = -21 \to 21$ $l = -24 \to 24$	$h = -18 \to 18$ $k = -12 \to 12$ $l = -20 \to 20$	$h = -18 \to 18$ $k = -11 \to 11$ $l = -22 \to 22$	$h = -17 \to 23$ $k = -10 \to 13$ $l = -27 \to 24$	$h = -9 \to 14$ $k = -16 \to 16$ $l = -17 \to 17$
No. of measured, independent and observed [$I > 2\sigma(I)$] reflections	62376, 11982, 8633	20912, 9227, 6586	70840, 18800, 12909	17655, 4810, 3816	15489, 4671, 3679	19934, 4407, 2963	14908, 8035, 7066
R_{int}.	0.065	0.029	0.072	0.029	0.026	0.068	0.019
Data / restraints / parameters	11982/8/642	9227/7/602	18800/6/993	4810/0/257	4671/1/257	4407/2/227	8035/4/370
$R[F^2 > 2\sigma(F^2)]$,[a] $wR(F^2)$,[b] S[c]	0.057, 0.127, 1.07	0.0388, 0.0915, 0.986	0.064, 0.160, 1.03	0.038, 0.112, 1.03	0.035, 0.093, 1.04	0.059, 0.136, 1.01	0.026, 0.059, 1.04
$(\Delta/\sigma)_{max}$	0.001	0.001	< 0.001	0.001	0.001	< 0.001	0.004
$\Delta\rho_{max}$, $\Delta\rho_{min}$/ e $Å^{-3}$	0.74, −0.39	0.041, −0.44	0.94, −0.71	1.00, −0.30	0.18, −0.39	0.51, −0.37	0.84, −0.70

[a] $R = \Sigma || F_o| - | F_c| | / \Sigma | F_o|$, [b] $wR = [\Sigma(F_o^2 - F_c^2)^2 / \Sigma w(F_o^2)^2]^{1/2}$, [c] $S = \Sigma[w(F_o^2 - F_c^2)^2/(N_{obs} - N_{param})]^{1/2}$

Table S2 Selected bond lengths [Å] and angles [°] for complex **1**·2MeOH·MeCN

**Complex**	**1**·2MeOH·MeCN
**Bond distances**	
Ni1−N11	1.994(3)
Ni1−O1	2.012(2)
Ni1−O12	2.046(2)
Ni1−O11	2.071(2)
Ni1−O13	2.076(3)
Ni1−O14	2.154(2)
Ni2−O2	2.020(2)
Ni2−O22	2.022(2)
Ni2−O12	2.022(2)
Ni2−O3	2.025(2)
Ni2−O15	2.098(2)
Ni2−O25	2.098(2)
Ni3−N21	1.990(3)
Ni3−O4	2.012(2)
Ni3−O21	2.024(2)
Ni3−O22	2.030(2)
Ni3−O24	2.114(3)
Ni3−O23	2.169(3)
**Bond angles**	
N11−Ni1−O1	170.65(10)
N11−Ni1−O12	90.57(10)
O1−Ni1−O12	98.68(9)
N11−Ni1−O11	78.47(10)
O1−Ni1−O11	92.48(9)
O12−Ni1−O11	167.44(9)
N11−Ni1−O13	89.87(10)
O1−Ni1−O13	88.46(10)
O12−Ni1−O13	91.79(10)
O11−Ni1−O13	94.28(10)
N11−Ni1−O14	94.29(10)
O1−Ni1−O14	87.87(10)
O12−Ni1−O14	85.37(9)
O11−Ni1−O14	89.31(9)
O13−Ni1−O14	174.98(9)
O2−Ni2−O22	87.42(9)
O2−Ni2−O12	101.90(9)
O22−Ni2−O12	167.79(9)
O2−Ni2−O3	88.97(10)

O22−Ni2−O3	102.89(9)
O12−Ni2−O3	85.34(9)
O2−Ni2−O15	178.80(11)
O22−Ni2−O15	91.98(9)
O12−Ni2−O15	78.55(9)
O3−Ni2−O15	92.18(11)
O2−Ni2−O25	93.39(10)
O22−Ni2−O25	78.75(9)
O12−Ni2−O25	92.69(9)
O3−Ni2−O25	177.20(10)
O15−Ni2−O25	85.47(11)
N21−Ni3−O4	167.90(10)
N21−Ni3−O21	78.82(10)
O4−Ni3−O21	89.94(10)
N21−Ni3−O22	90.92(10)
O4−Ni3−O22	100.76(9)
O21−Ni3−O22	167.60(10)
N21−Ni3−O24	88.87(11)
O4−Ni3−O24	87.16(10)
O21−Ni3−O24	92.52(10)
O22−Ni3−O24	94.24(10)
N21−Ni3−O23	95.59(10)
O4−Ni3−O23	88.69(10)
O22−Ni3−O23	84.56(9)
O24−Ni3−O23	175.39(9)

Table S3. Hydrogen bonds and interactions geometry (Å,°)

**D-H···A**	***d*(D-H)**	***d*(H···A)**	***d*(D···A)**	**<(DHA)**	**Symmetry code**
**1**·2MeOH·MeCN					
C6−H36C···O1ME	0.98	2.42	3.383 (6)	166	-x+1/2, y-1/2, -z+1/2
C112−H112···N1	0.95	2.69	3.625 (6)	170	x-1, y, z
C117−H17D···O22	0.98	2.58	3.210 (5)	122	-
C217−H17A···O12	0.98	2.52	3.165 (5)	123	-
C217−H17B···N1	0.98	2.70	3.443 (6)	133	-
O1ME−H1ME···O21	0.83 (2)	1.90 (2)	2.718 (4)	170 (5)	-
O2ME−H2ME···O11	0.83 (2)	1.87 (2)	2.707 (4)	177 (5)	-x+1, -y+2, -z
O13−H13O···N22	0.84 (2)	1.91 (2)	2.738 (4)	170 (5)	x+1/2, -y+3/2, z-1/2
O14−H14O···O3	0.81 (2)	1.92 (2)	2.728 (3)	178 (5)	-
O16−H16O···O2ME	0.85 (2)	1.81 (2)	2.636 (5)	167 (5)	-
O23−H23O···O2	0.85 (2)	1.85 (2)	2.702 (3)	171 (5)	-
O24−H24O···N12	0.83 (2)	1.95 (2)	2.770 (4)	172 (5)	x-1/2, -y+3/2, z+1/2
O26−H26O···O1ME	0.84 (2)	1.85 (2)	2.682 (5)	169 (5)	-x-1, -y+2, -z+1
**2**·4.7MeOH					
C2-H2A···O4ME	0.98	2.694	3.492(4)	139	-
C4ME –H4MB···N12	0.98	2.615	3.376(3)	135	-
C111-H111···O21	0.95	2.351	3.214(3)	151	-
C116-H11E···O23	0.98	2.793	3.696(5)	153	-
C116-H11E···O4ME	0.98	2.558	3.357(5)	139	x+1,+y,+z
C211-H211···O11	0.95	2.328	3.206(3)	153	-
O3ME-H3ME···O2	0.81(3)	1.92(3)	2.726(3)	176(2)	-x+1,-y+1,-z+1
O4ME-H4ME···O1	0.84(3)	1.86(3)	2.710(3)	177(3)	-
C3ME-H3MB···O24	0.98	2.876	3.603(4)	132	-x+2,-y+1,-z+1
C2-H2A···O23	0.98	2.98	3.760(4)	138	x-1,+y,+z
C1ME-H1MA···O13	0.98	2.96	3.680(6)	132	-x+1,-y+1,-z
C2ME-H2MA···O5MA	0.98	2.53	3.427(7)	153	x+1,+y,+z
C216-H21F···O24	0.98	2.69	3.549(4)	146	-x+2,-y+1,-z+1
C27-H27···O3ME	0.95	2.49	3.387(3)	157	-
C28-H28···O3ME	0.95	2.63	3.402(4)	139	-
N22-H22···O3ME	0.91(4)	1.93(3)	2.819(3)	167(3)	-
C4ME-H4MA···O15	0.98	2.41	3.230(4)	141	-x+1,-y,-z+1
C17-H17···O1ME	0.95	2.96	3.662(4)	132	x,+y-1,+z
C17-H17···O2ME	0.95	2.56	3.459(3)	157	x,+y-1,+z
C14-H14···O24	0.95	2.64	3.283(4)	126	x-1,+y,+z-1
C18-H18···O2ME	0.95	2.57	3.374(4)	142	x,+y-1,+z
N12-H12···O2ME	0.87(4)	2.04(3)	2.894(3)	166(3)	x,+y-1,+z

C17-H17···O2ME	0.95	2.56	3.459(3)	157	x,+y-1,+z
C24-H24A···O5MB	0.95	2.73	3.679(7)	176	-x+1,-y+1,-z+1
O24-H24···O14	0.86(2)	1.70(2)	2.559(2)	173(2)	x+1,+y,+z+1
C116-H11E···O4ME	0.98	2.56(1)	3.357(5)	139	x+1,+y,+z
O23-H23···O4ME	0.75(3)	1.90(3)	2.635(3)	168(4)	x+1,+y,+z
O13-H13···O25	0.75(3)	2.06(3)	2.785(2)	164(3)	-x+1,-y+1,-z
O2ME-H2ME···O1ME	0.81(4)	1.84(4)	2.647(5)	173(3)	-
O1ME-H1ME···O14	0.85(4)	1.85(4)	2.696(4)	176(4)	-x,-y+1,-z
O5MA-H5MA···O14	0.84(1)	1.96(1)	2.797(4)	172(1)	-x,-y+1,-z
O2ME-H2ME···O5MB	0.81(4)	2.25(3)	2.865(7)	133(3)	-
C17-H17···O5MB	0.95	2.88	3.641(7)	138	x,+y-1,+z
C24-H24A···O14	0.95	2.74	3.355(3)	123	x,+y-1,+z
**3**·4MeOH·0.63H_2O·0.5MeCN·HOAc					
O2ME-H2ME···O1W	0.8400	2.00	2.756(11)	150	-
N32-H3N···O1W	0.87(4)	1.98(5)	2.803(6)	157(5)	-1+x,y,z
O4ME-H4ME···N12	0.84	2.03	2.840(5)	161	x,1+y,z
N42-H4N···O5ME	0.89(5)	2.01(5)	2.848(4)	156(6)	-
O6-H6···O2ME	0.84	2.02	2.721(14)	140	1-x,1-y,-z
O5ME-H5ME···O3	0.84	1.89	2.726(4)	172	1-x,1-y,1-z
O13-H13O···O4	0.86(5)	1.86(5)	2.712(4)	174(5)	1+x,y,z
O23-H23O···O1	0.86(4)	1.88(5)	2.681(4)	154(5)	-x,1-y,-z
O33-H33O···O5	0.85(4)	2.00(5)	2.764(6)	148(7)	x,-1+y,z
O43-H43O···O4ME	0.84(6)	1.77(6)	2.559(5)	156(8)	-
C211-H026···O11	0.95	2.25	3.117(5)	152	-
C2-H2A···O23	0.98	2.52	3.492(6)	171	1+x,y,z
C2ME-H8B···O6	0.98	2.49	3.21(2)	130	1+x,-1+y,z
C2ME-H8B···O2ME	0.98	2.37	3.26(2)	151	2-x,-y,-z
C100-H10D···N5	0.98	2.41	3.24(2)	141	-
C16-H16···O13	0.95	2.30	3.244(6)	174	2-x,-y,1-z
C18-H18···O43	0.95	2.48	3.357(6)	153	x,-1+y,z
C38-H38···O1W	0.95	2.59	3.312(6)	133	-1+x,y,z
C47-47···O5ME	0.95	2.52	3.435(5)	162	-
C111-H111···O21	0.95	2.29	3.162(5)	153	-
C312-H312···O41	0.95	2.27	3.150(4)	154	-
C411-H411···O31	0.95	2.30	3.186(4)	154	-

**4**					
O4−H4O···N2	0.72(3)	2.07(3)	2.783(2)	172(3)	-x+1/2+1,+y-1/2,-z-1/2
C15−H15···O3	0.93	2.93	3.855(3)	174(1)	-
C6−H6···N2	0.93	2.69	3.352(3)	129(1)	-x+1/2+1,+y-1/2,-z-1/2
C6−H6···O3	0.93	2.99	3.763(3)	142(1)	x+1/2,-y+1/2,+z-1/2
C8−H8···O4	0.93	2.82	3.546(3)	136(1)	-x+1/2+1,+y+1/2,-z-1/2
C7−H7···O4	0.93	2.54	3.431(3)	160(1)	-x+1/2+1,+y+1/2,-z-1/2
**5**					
O4−H4O···N2	0.83 (2)	2.88 (2)	3.638 (2)	152 (2)	-x+1/2, y+1/2, -z-1/2
C15−H15···O1	0.93	2.24	2.796 (2)	118	
C19−H19···O2	0.93	2.19	2.721 (2)	116	
C16−H16···O3	0.93	2.48	3.389 (3)	164	x-1/2, -y+3/2, z-1/2
C15−H15···O1	0.93	2.24	2.796 (2)	118	
C19−H19···O2	0.93	2.19	2.721 (2)	116	
C16−H16···O3	0.93	2.48	3.389 (3)	164	x-1/2, -y+3/2, z-1/2
**9**·2MeOH					
C8−H8···O4	0.95	2.57	3.350 (3)	140	-
C7−H7···O4	0.95	2.47	3.374(4)	159	-
O3−H3O···O2	0.92 (4)	1.59 (4)	2.506 (2)	175 (4)	*x*, -*y*+1, *z*-1/2
N2−H2···O4	0.92 (4)	1.90 (4)	2.808 (3)	170 (4)	
O4−H4O···O5	0.86 (2)	1.80 (2)	2.620 (4)	159 (4)	
O5−H5O···O3	0.84(5)	1.88(5)	2.688(4)	160(1)	x+1/2,-y+1/2,-z
C11−H11···O3	0.95	2.66	3.279(4)	124	x,-y+1,+z+1/2
C13−H13···O1	0.95	2.62	3.528(3)	160	x+1/2,+y+1/2,-z+1/2
C15−H15A···O4	0.98	2.96	3.873(6)	155	-x+1/2,+y+1/2,+z
C16−H16A···O2	0.98	2.88	3.781(5)	153	x+1/2,+y-1/2,-z+1/2
**10**					
N2-H2N···O12	0.86(2)	2.03(2)	2.866(2)	163(2)	-
C3-H3···O12	0.95	2.89	3.555(2)	128	-
C8-H8···O11	0.95	2.89	3.773(2)	156	-
C8-H8···O12	0.95	2.68	3.439(3)	137	-
C14-H14···O11	0.95	2.89	3.773(3)	160	-
C20-H20C···O8	0.98	2.66	3.389(3)	132	-
O4-H4O···O11	0.78(2)	1.97(2)	2.743(2)	173(3)	-x+2,-y+1,-z+2
C3-H3···O6	0.95	2.98	3.729(3)	137	-x+2,-y+1,-z+2
O5-H5O···O8	0.80(2)	1.87(2)	2.671(2)	176(2)	-x+2,-y,-z+1
C4-H4···O8	0.95	2.70(1)	3.352(3)	127	-x+2,-y,-z+1
C11-H11···O1	0.95	2.34	3.236(3)	156	-x+1,-y+1,-z+2
C16-H16A···O3	0.98	2.79	3.472(3)	127	-x+1,-y+1,-z+2

C13-H13···O6	0.95	2.67	3.303(3)	125	-x+2,-y+2,-z+2
C15-H15A···O8	0.98	2.70	3.432(3)	132	-x+2,-y+1,-z+1
C17-H17B···O7	0.98	2.39	3.353(3)	167	x,+y+1,+z+1
O3-H3O···O13	0.78(2)	1.99(2)	2.764(2)	169(2)	x-1,+y,+z

Table S4. Selected bond lengths [Å] and angles [°] for complex **2**·4.7MeOH**.**

**Complex**	**2**·4.7MeOH
**Bond distances**	
Ni1−N11	1.997(2)
Ni1−O12	2.0194(15)
Ni1−O22	2.0390(16)
Ni1−O1	2.0643(16)
Ni1−O11	2.0953(16)
Ni1−O13	2.0970(18)
Ni2−N21	1.995(2)
Ni2−O22	2.0320(16)
Ni2−O12	2.0326 15)
Ni2−O2	2.0611(16)
Ni2−O23	2.0883(17)
Ni2−O21	2.1028(16)
**Bond angles**	
N11−Ni1−O12	89.66(7)
N11−Ni1−O22	172.10(7)
O12−Ni1−O22	82.74(6)
N11−Ni1−O1	94.21(7)
O12−Ni1−O1	90.58(6)
O22−Ni1−O1	88.01(6)
N11−Ni1−O11	78.19(7)
O12−Ni1−O11	167.80(6)
O22−Ni1−O11	109.37(6)
O1−Ni1−O11	91.38(7)
N11−Ni1−O13	87.64(8)
O12−Ni1−O13	94.86(7)
O22−Ni1−O13	90.70(10)
O1−Ni1−O13	174.27(7)
O11−Ni1−O13	83.68(7)
N21−Ni2−O22	90.12(7)
N21−Ni2−O12	172.27(7)
O22−Ni2−O12	82.59(6)
N21−Ni2−O2	93.74(7)
O22−Ni2−O2	90.27(6)
O12−Ni2−O2	88.87(6)
N21−Ni2−O23	88.16(8)
O22−Ni2−O23	95.08(7)
O12−Ni2−O23	89.94(7)
O2−Ni2−O23	174.33(7)

N21−Ni2−O21	78.00(7)
O22−Ni2−O21	168.10(6)
O12−Ni2−O21	109.31(6)
O2−Ni2−O21	89.72(7)
O23−Ni2−O21	85.44(7)

Table S5. Selected bond lengths [Å] and angles [°] for complex **3**·4MeOH·0.63H_2O·0.5MeCN·HOAc

**Complex**	**3**·4MeOH·0.63H_2O·0.5MeCN·HOAc
**Bond distances**	
Ni1−N11	1.980 (3)
Ni1−O11	2.006 (2)
Ni1−O2	2.041 (3)
Ni1−O12	2.074 (2)
Ni1−O22	2.091 (2)
Ni1−O42	2.172 (2)
Ni2−N21	1.997 (3)
Ni2−O1	2.035 (3)
Ni2−O21	2.038 (3)
Ni2−O22	2.073 (2)
Ni2−O12	2.077 (2)
Ni2−O32	2.140 (3)
Ni3−N31	2.000 (3)
Ni3−O4	2.058 (1)
Ni3−O42	2.061 (2)
Ni3−O32	2.065 (2)
Ni3−O31	2.069 (3)
Ni3−O12	2.120 (3)
Ni4−N41	1.998 (3)
Ni4−O42	2.043 (2)
Ni4−O3	2.051 (3)
Ni4−O41	2.052 (2)
Ni4−O32	2.069 (2)
Ni4−O22	2.113 (2)
**Bond angles**	
N11−Ni1−O11	79.22 (11)
N11−Ni1−O2	93.59 (12)
O11−Ni1−O2	98.21 (10)
N11−Ni1−O12	91.26 (10)
O11−Ni1−O12	167.10 (10)
O2−Ni1−O12	91.00 (10)
N11−Ni1−O22	176.74 (11)
O11−Ni1−O22	102.01 (10)
O2−Ni1−O22	89.20 (10)
O12−Ni1−O22	87.05 (9)

N11−Ni1−O42	95.17 (11)
O11−Ni1−O42	91.92 (10)
O2−Ni1−O42	167.68 (10)
O12−Ni1−O42	80.19 (9)
O22−Ni1−O42	81.80 (9)
N21−Ni2−O1	94.44 (11)
N21−Ni2−O21	77.82 (11)
O1−Ni2−O21	97.70 (10)
N21−Ni2−O22	90.14 (10)
O1−Ni2−O22	91.40 (10)
O21−Ni2−O22	165.39 (10)
N21−Ni2−O12	174.66 (12)
O1−Ni2−O12	90.39 (10)
O21−Ni2−O12	103.82 (10)
O22−Ni2−O12	87.46 (9)
N21−Ni2−O32	93.36 (11)
O1−Ni2−O32	168.67 (10)
O21−Ni2−O32	91.98 (10)
O22−Ni2−O32	80.37 (9)
O12−Ni2−O32	81.54 (10)
N31−Ni3−O4	94.15 (11)
N31−Ni3−O42	173.65 (11)
O4−Ni3−O42	89.34 (10)
N31−Ni3−O32	88.40 (11)
O4−Ni3−O32	88.98 (10)
O42−Ni3−O32	86.36 (9)
N31−Ni3−O31	78.07 (11)
O4−Ni3−O31	97.76 (10)
O42−Ni3−O31	106.74 (10)
O32−Ni3−O31	165.24 (10)
N31−Ni3−O12	94.02 (10)
O4−Ni3−O12	167.86 (10)
O42−Ni3−O12	81.73 (9)
O32−Ni3−O12	82.29 (10)
O31−Ni3−O12	92.71 (10)
N41−Ni4−O42	89.52 (10)
N41−Ni4−O3	93.68 (11)
O42−Ni4−O3	90.10 (10)
N41−Ni4−O41	77.86 (11)
O42−Ni4−O41	165.85 (9)
O3−Ni4−O41	97.00 (10)
N41−Ni4−O32	175.28 (11)

O42−Ni4−O32	86.74 (9)
O3−Ni4−O32	89.21 (10)
O41−Ni4−O32	105.50 (10)
N41−Ni4−O22	95.66 (11)
O42−Ni4−O22	84.40 (9)
O3−Ni4−O22	169.11 (10)
O41−Ni4−O22	90.44 (10)
O32−Ni4−O22	81.11 (9)

Table S6. Selected bond lengths [Å] and angles [°] for complexes: **4**, **5** and **9**·2MeOH

**Complex**	**4**	**5**	**9**·2MeOH
**Bond length**			
Ni1−O2	1.8149 (14)	1.8164 (13)	2.0398 (18)
Ni1−N1	1.8323 (17)	1.8339 (14)	2.011 (2)
Ni1−O1	1.8444 (14)	1.8443 (12)	2.0983 (18)
Ni1−O2^{i}			2.0398 (18)
Ni1−N1^{i}			2.011 (2)
Ni1−O1^{i}			2.0983 (18)
Ni1−N3	1.9452 (17)	1.9453 (15)	
Bond angles			
O2−Ni1−N1	95.14 (7)	95.39 (6)	88.28 (8)
O2−Ni1−O1	178.20 (6)	178.49 (5)	166.39 (7)
N1−Ni1−O1	83.07 (7)	83.39 (6)	78.46 (8)
O2−Ni1−N3	89.58 (7)	89.08 (6)	
N1−Ni1−N3	175.28 (7)	175.28 (6)	
O1−Ni1−N3	92.21 (7)	92.16 (6)	
N1−Ni1−N1^{i}			173.70 (13)
N1i−Ni1−O2			95.99 (8)
N1−Ni1−O2^{i}			96.00 (8)
N1i−Ni1−O2^{i}			88.28 (8)
O2−Ni1−O2^{i}			94.59 (11)
N1−Ni1−O1^{i}			96.97 (8)
N1i−Ni1−O1^{i}			78.46 (8)
O2−Ni1−O1^{i}			89.93 (7)
O2i−Ni1−O1^{i}			166.38 (7)
N1i−Ni1−O1			96.97 (8)
O2i−Ni1−O1			89.93 (7)
O1i−Ni1−O1			88.61 (10)

Symmetry code: (i) -*x*, *y*, -*z*+1/2 for complex **9**·2MeOH

Table S7. Selected bond lengths [Å] and angles [°] for complex **10**

**Complex**	**10**
**Bond distances**	
Ni1−N1	1.9936 (16)
Ni1−O2	2.0254 (13)
Ni1−O2^{i}	2.0262 (13)
Ni1−O1	2.0919 (14)
Ni1−O4	2.0986 (15)
Ni1−O3	2.1047 (14)
Mo1−O7	1.7021 (14)
Mo1−O8	1.7127 (14)
Mo1−O14^{ii}	1.8624 (13)
Mo1−O10	2.0512 (13)
Mo1−O9	2.2182 (13)
Mo1−O9^{ii}	2.3614 (13)
Mo2−O12	1.7103 (14)
Mo2−O11	1.7112 (14)
Mo2−O13	1.9316 (14)
Mo2−O14	1.9830 (13)
Mo2−O10	2.2114 (13)
Mo2−O9	2.2457 (13)
**Bond angles**	
N1−Ni1−O2	89.46 (6)
N1−Ni1−O2^{i}	169.84 (6)
O2−Ni1−O2^{i}	80.50 (5)
N1−Ni1−O1	78.69 (6)
O2−Ni1−O1	168.13 (5)
O2^{i}−Ni1−O1	111.32 (5)
N1−Ni1−O4	85.04 (7)
O2−Ni1−O4	92.17 (6)
O2^{i}−Ni1−O4	93.71 (6)
O1−Ni1−O4	86.16 (6)
N1−Ni1−O3	94.28 (6)
O2−Ni1−O3	93.93 (6)
O2^{i}−Ni1−O3	88.02 (6)
O1−Ni1−O3	87.72 (6)
O4−Ni1−O3	173.86 (6)
O7−Mo1−O8	105.91 (8)

O7−Mo1−O14^{ii}	103.61 (6)
O8−Mo1−O14^{ii}	101.22 (6)
O7−Mo1−O10	91.03 (6)
O8−Mo1−O10	98.29 (6)
O14^{ii}−Mo1−O10	151.20 (6)
O7−Mo1−O9	154.43 (6)
O8−Mo1−O9	95.31 (6)
O14^{ii}−Mo1−O9	85.64 (5)
O10−Mo1−O9	71.59 (5)
O7−Mo1−O9^{ii}	87.59 (6)
O8−Mo1−O9^{ii}	166.33 (6)
O14^{ii}−Mo1−O9^{ii}	72.87 (5)
O10−Mo1−O9^{ii}	83.31 (5)
O9−Mo1−O9^{ii}	72.22 (5)
O12−Mo2−O11	107.37 (7)
O12−Mo2−O13	100.47 (6)
O11−Mo2−O13	94.00 (7)
O12−Mo2−O14	95.17 (6)
O11−Mo2−O14	95.38 (6)
O13−Mo2−O14	158.45 (6)
O12−Mo2−O10	90.51 (6)
O11−Mo2−O10	162.04 (6)
O13−Mo2−O10	84.09 (6)
O14−Mo2−O10	81.02 (5)
O12−Mo2−O9	157.00 (6)
O11−Mo2−O9	93.83 (6)
O13−Mo2−O9	86.50 (5)
O14−Mo2−O9	73.55 (5)
O10−Mo2−O9	68.25 (5)

Symmetry codes: (i) -x+1, -y+1, -z+2; (ii) -x+2, -y+1, -z+1.

Powder X-ray diffraction

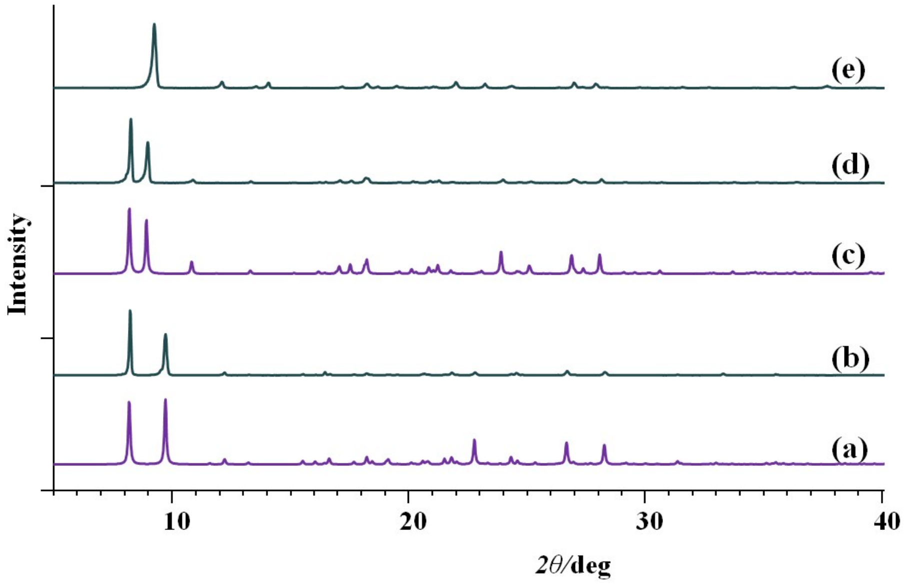

Figure S7. PXRD patterns of the mononuclear nickel(II) complexes: (a and b) [Ni(L^{3OMe})(py)] (**4**), (c and d) [Ni(L^{4OMe})(py)] (**5**), and (e) [Ni(L^{H})(py)] (**6**). The dark green lines indicate patterns obtained by powder diffraction, while the purple lines indicate patterns calculated from the X-ray single-crystal structures of the corresponding compounds.

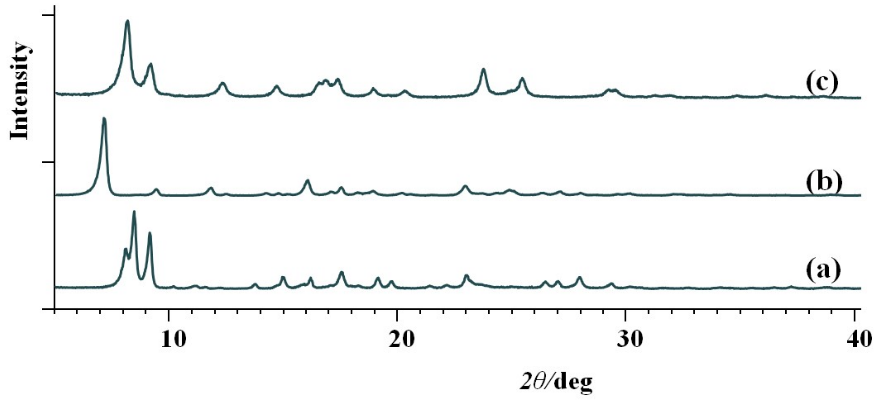

Figure S8. PXRD patterns of mononuclear nickel(II) complexes (a) [Ni(HL^{4OMe})$_2$] (**7**), (b) [Ni(HL^{4OMe})$_2$] (**8**), and (c) [Ni(HL^{H})$_2$] (**9**) .

Thermal analysis

Samples of $[Ni_3(L^{3OMe})_2(OAc)_2(MeOH)_2]\cdot 2MeOH\cdot MeCN$ (**1**·2MeOH·MeCN), $[Ni_2(HL^{4OMe})(L^{4OMe})(OAc)(MeOH)_2]\cdot 4.7MeOH$ (**2**·4.7MeOH), and $[Ni_4(HL^{H})_2(L^{H})_2(OAc)_2]\cdot 4MeOH\cdot 0.63H_2O\cdot 0.5MeCN\cdot HOAc$ (**3**·4MeOH·0.63H₂O·0.5MeCN·HOAc), $[Ni(HL^{3OMe})_2]\cdot MeOH$ (**7**·MeOH), $[Ni(HL^{H})_2]\cdot 2MeOH$ (**9**·2MeOH), and $[Ni_2(HL^{4OMe})_2(CH_3OH)_4][Mo_4O_{10}(OCH_3)_6]$ (**10**) were dried in a desiccator (at −15 °C) up to the constant weight prior to analysis.

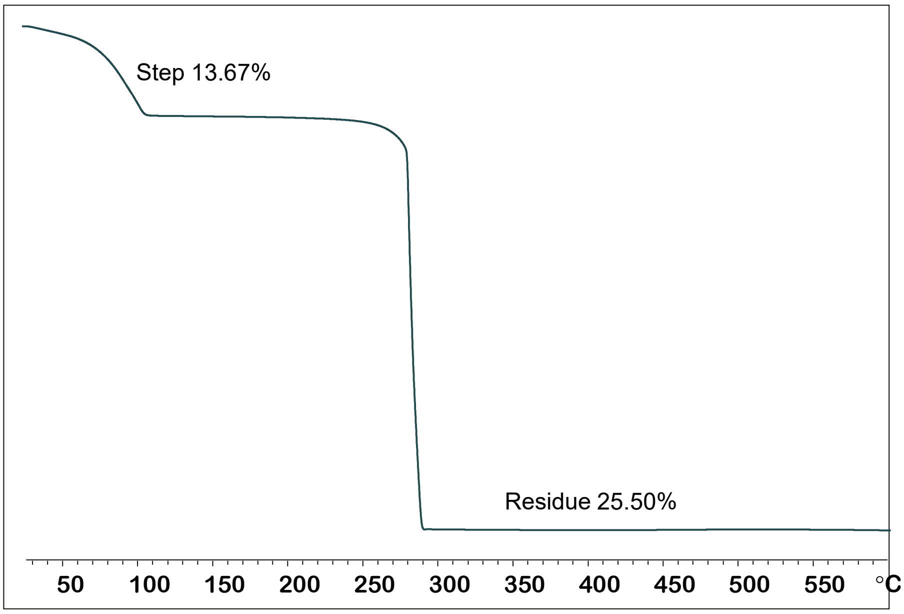

Lab: METTLER

Figure S9 TGA thermogram of desolvated acetato and phenoxido bridged trinuclear nickel(II) cluster $[Ni_3(L^{3OMe})_2(OAc)_2]$.

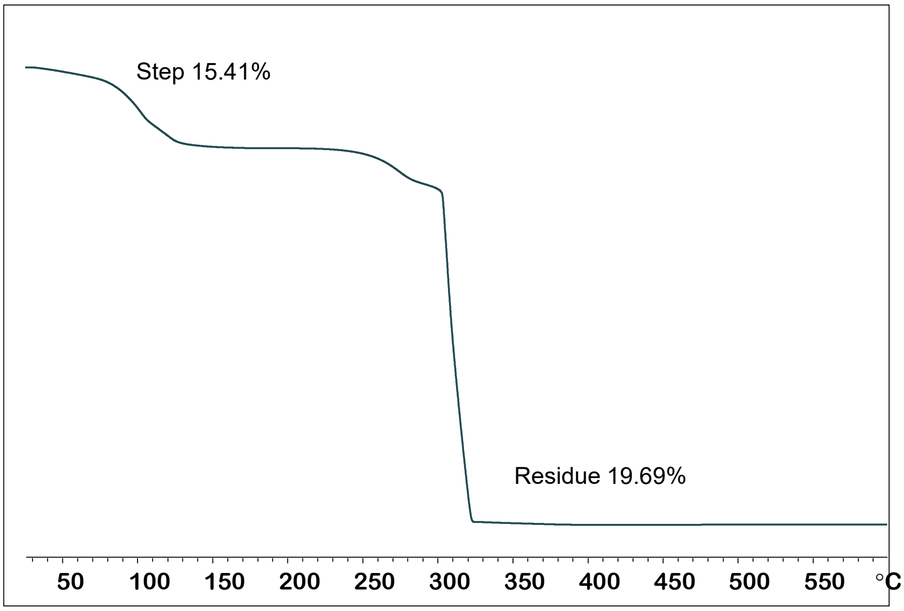

Lab: METTLER

Figure S10 TGA thermogram of desolvated acetato and phenoxido bridged dinuclear nickel(II) cluster $[Ni_2(HL^{4OMe})(L^{4OMe})(OAc)_2]$.

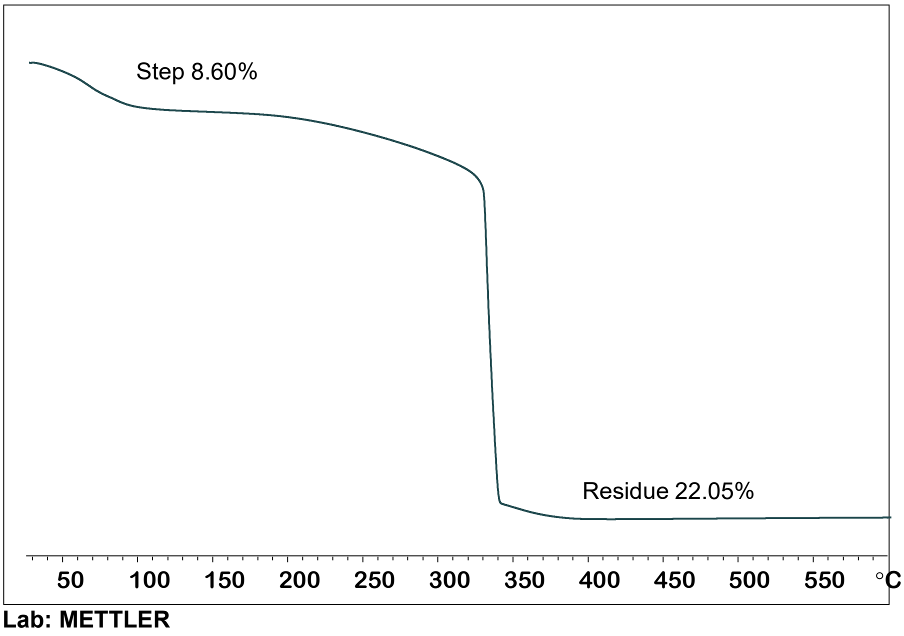

Figure S11 TGA thermogram of desolvated acetato and phenoxido bridged tetranuclear nickel(II) cluster $[Ni_4(HL^H)_2(L^H)_2(OAc)_2]$.

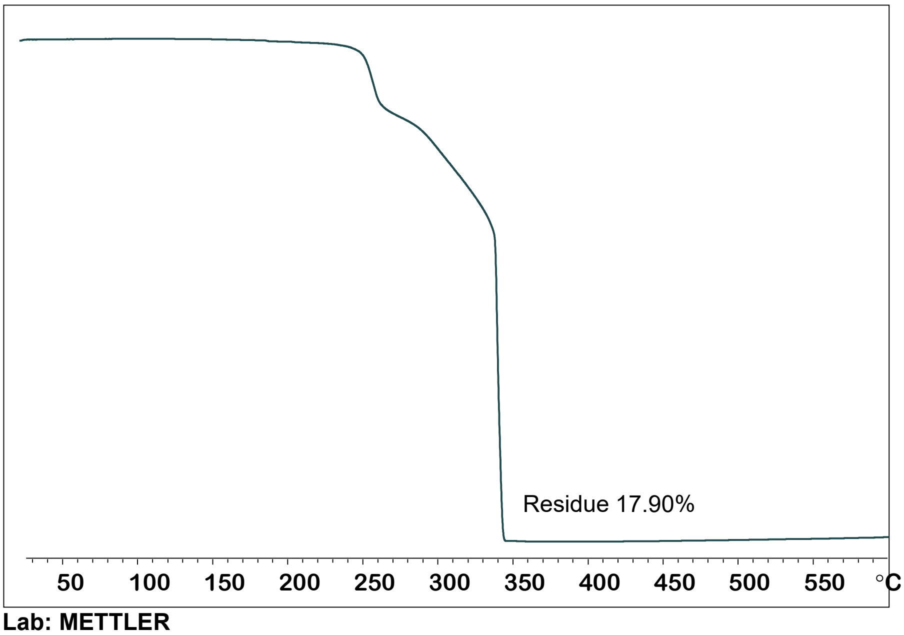

Figure S12 TGA thermogram of mononuclear nickel(II) complex $[Ni(L^{3OMe})(py)]$ (**4**).

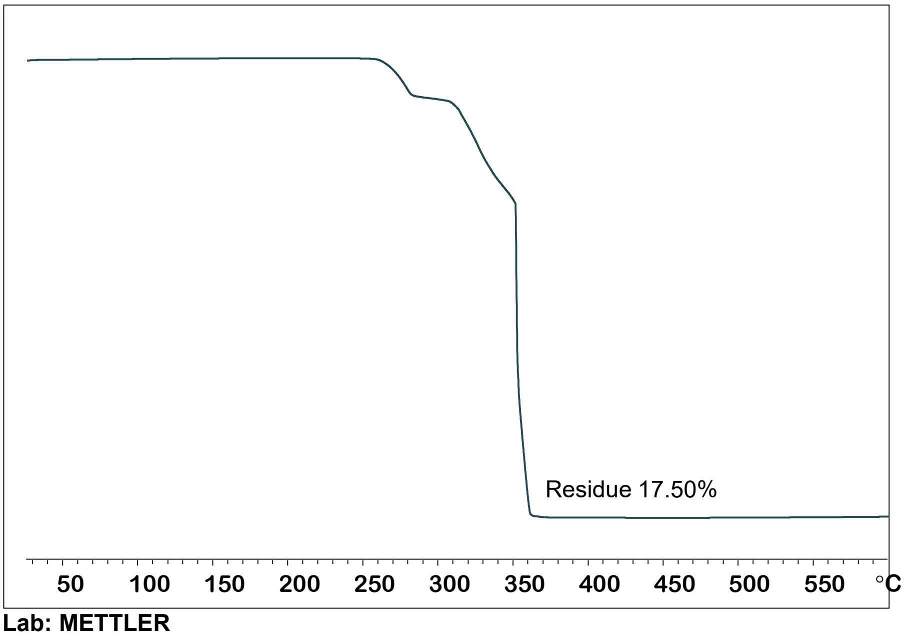

Figure S13 TGA thermogram of mononuclear nickel(II) complex [Ni(L^{4OMe})(py)] (**5**).

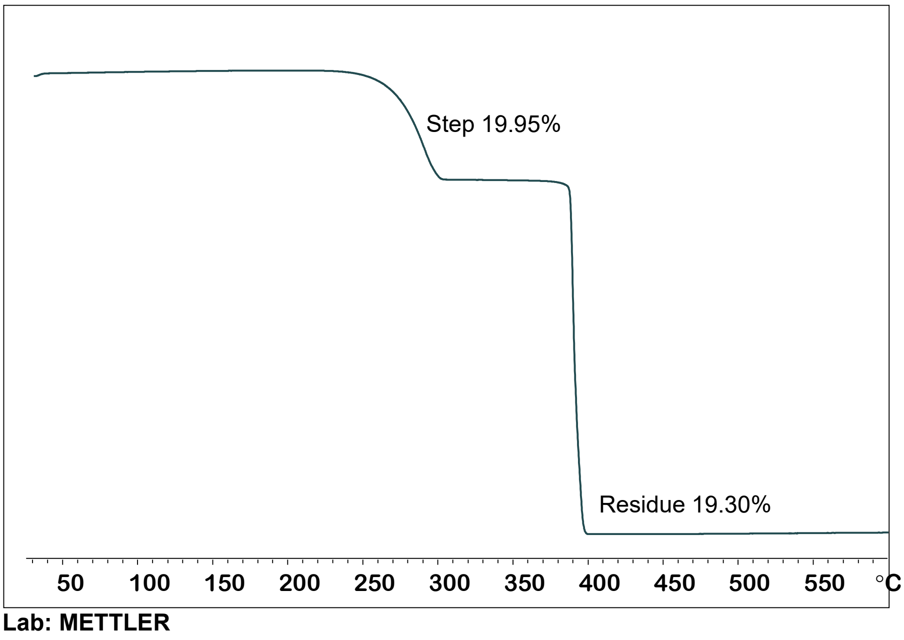

Figure S14 TGA thermogram of mononuclear nickel(II) complex [Ni(L^{H})(py)] (**6**).

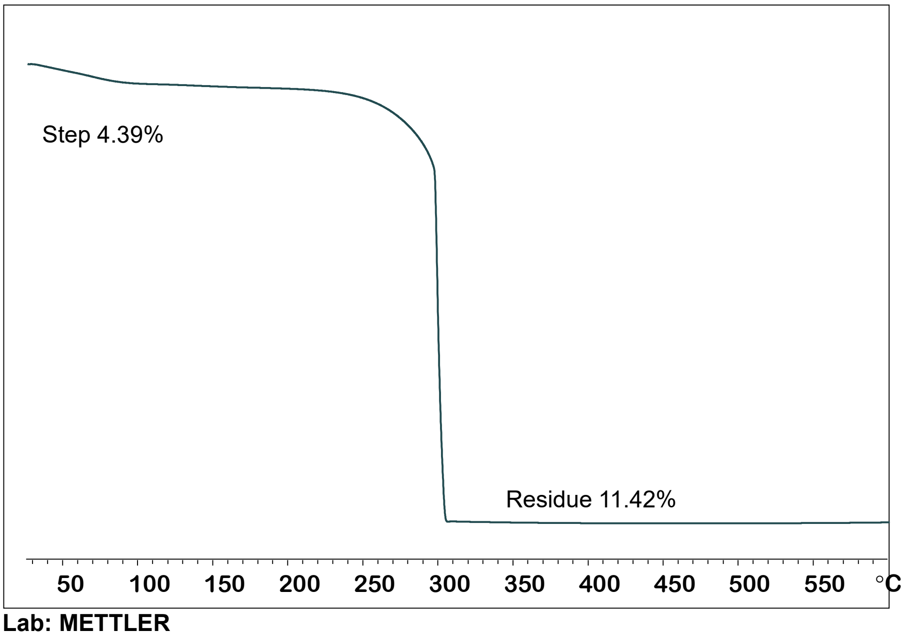

Figure S15 TGA thermogram of mononuclear nickel(II) complex [Ni(HL^{3OMe})$_2$]·MeOH (**7**·MeOH).

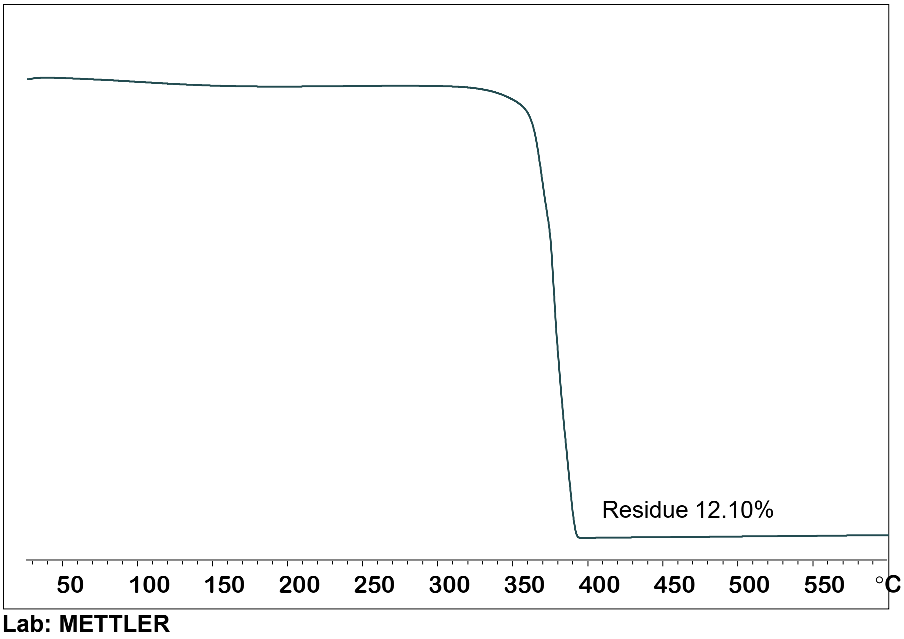

Figure S16 TGA thermogram of desolvated mononuclear nickel(II) complex [Ni(HL^{4OMe})$_2$] (**8**).

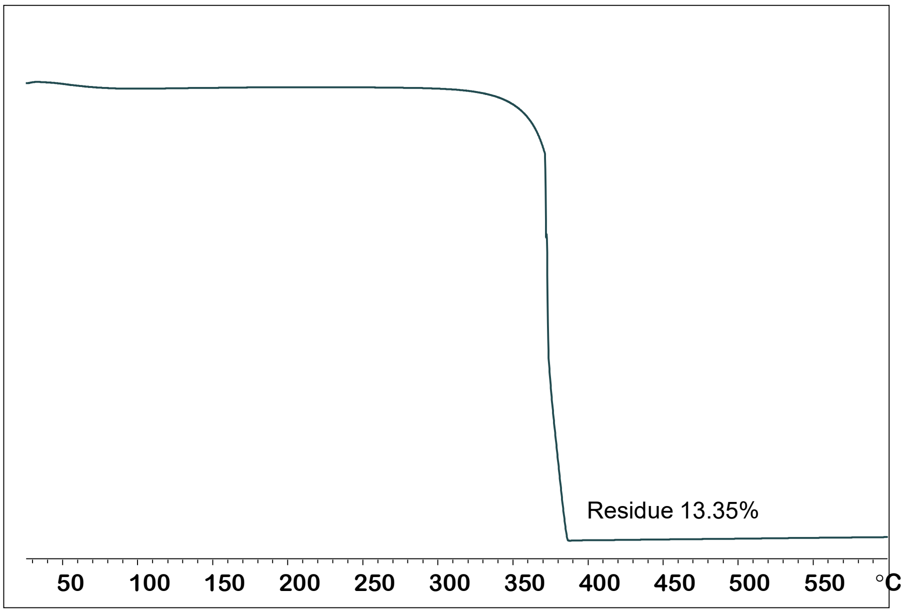

Lab: METTLER

Figure S17 TGA thermogram of desolvated mononuclear nickel(II) complex $[Ni(HL^{H})_2]$ (**9**).

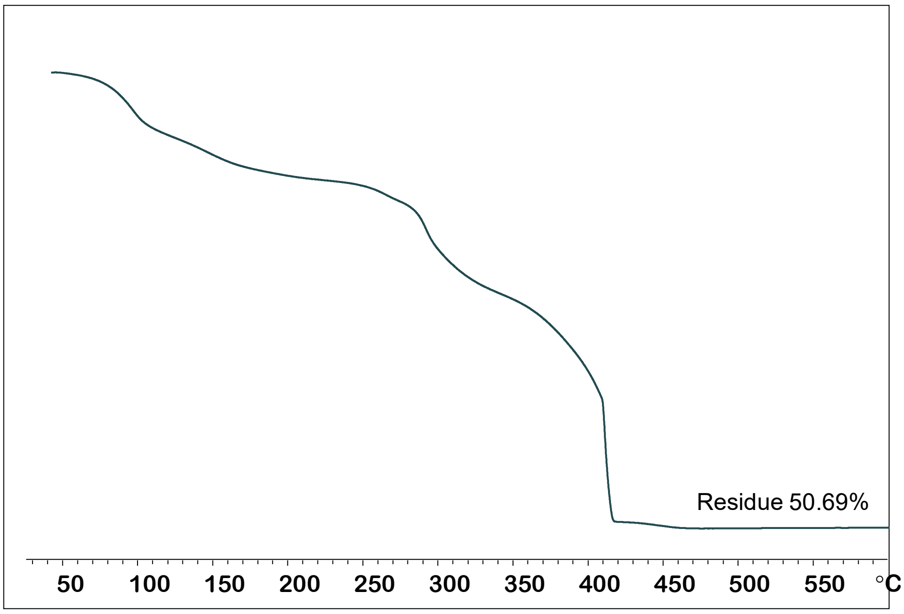

Lab: METTLER

Figure S18 IR spectra of desolvated hybrid organic-inorganic compound based on polyoxomolybdate $[Ni_2(HL^{4OMe})_2][Mo_4O_{10}(OCH_3)_6]$.

IR spectroscopy

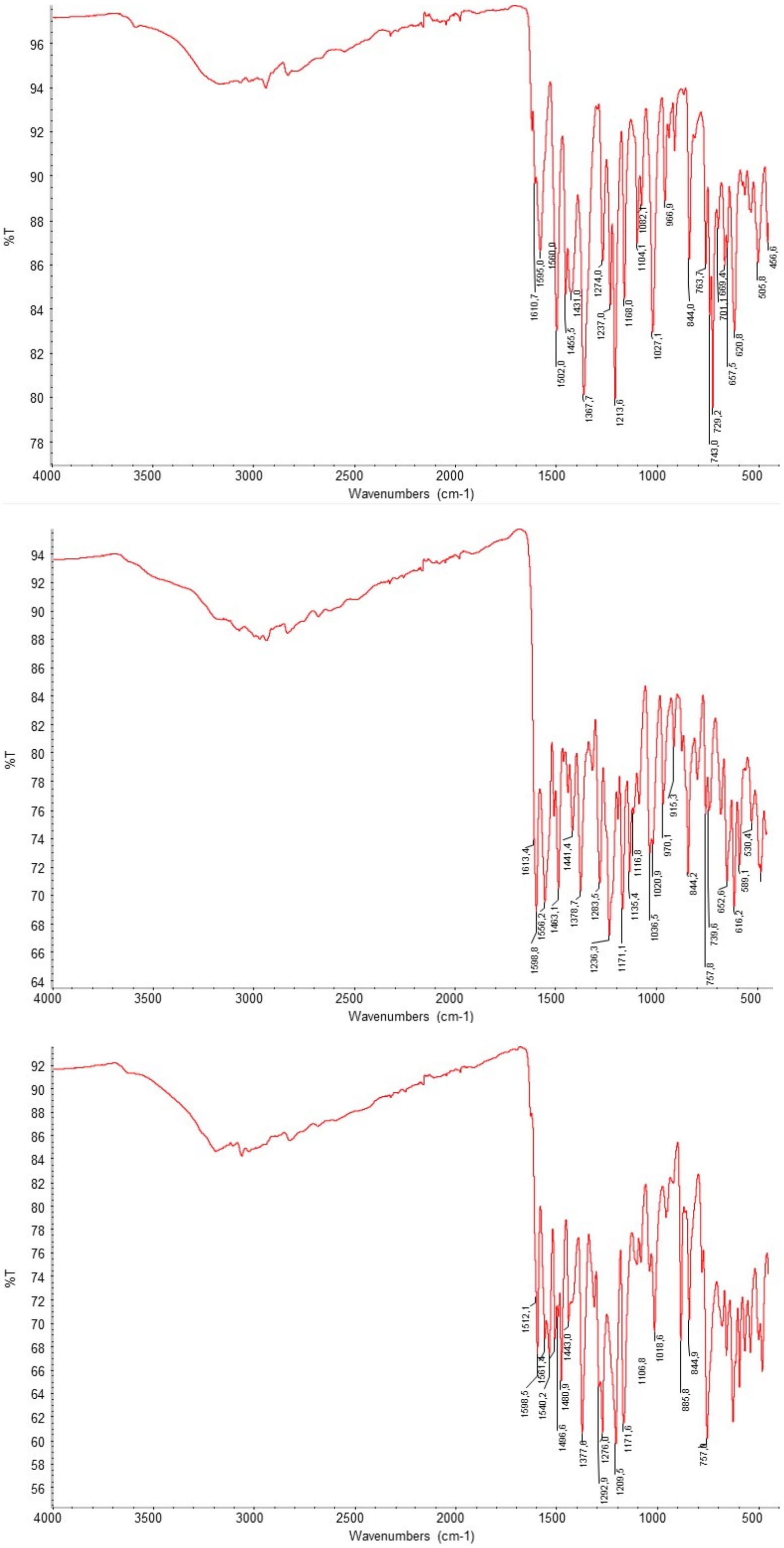

Figure S19 IR spectra of acetato and phenoxido bridged tri-, di-, and tetranuclear nickel(II) clusters: $[Ni_3(L^{3OMe})_2(OAc)_2]$, $[Ni_2(HL^{4OMe})(L^{4OMe})(OAc)_2]$, and $[Ni_4(HL^{H})_2(L^{H})_2(OAc)_2]$ (from top to bottom).

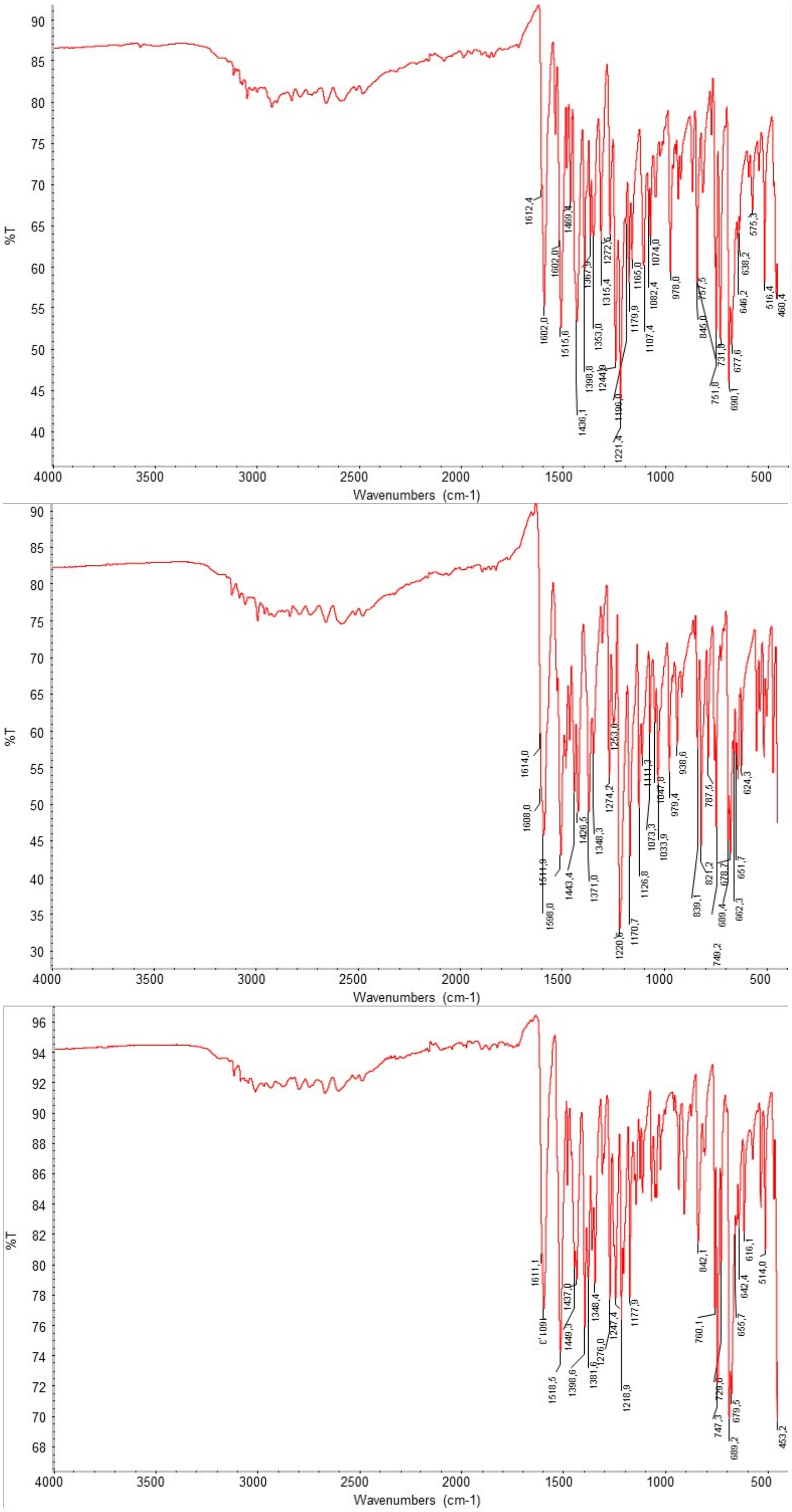

Figure S20 IR spectra of mononuclear nickel(II) complexes [Ni(L)(py)] (**4-6**) **(**from top to bottom).

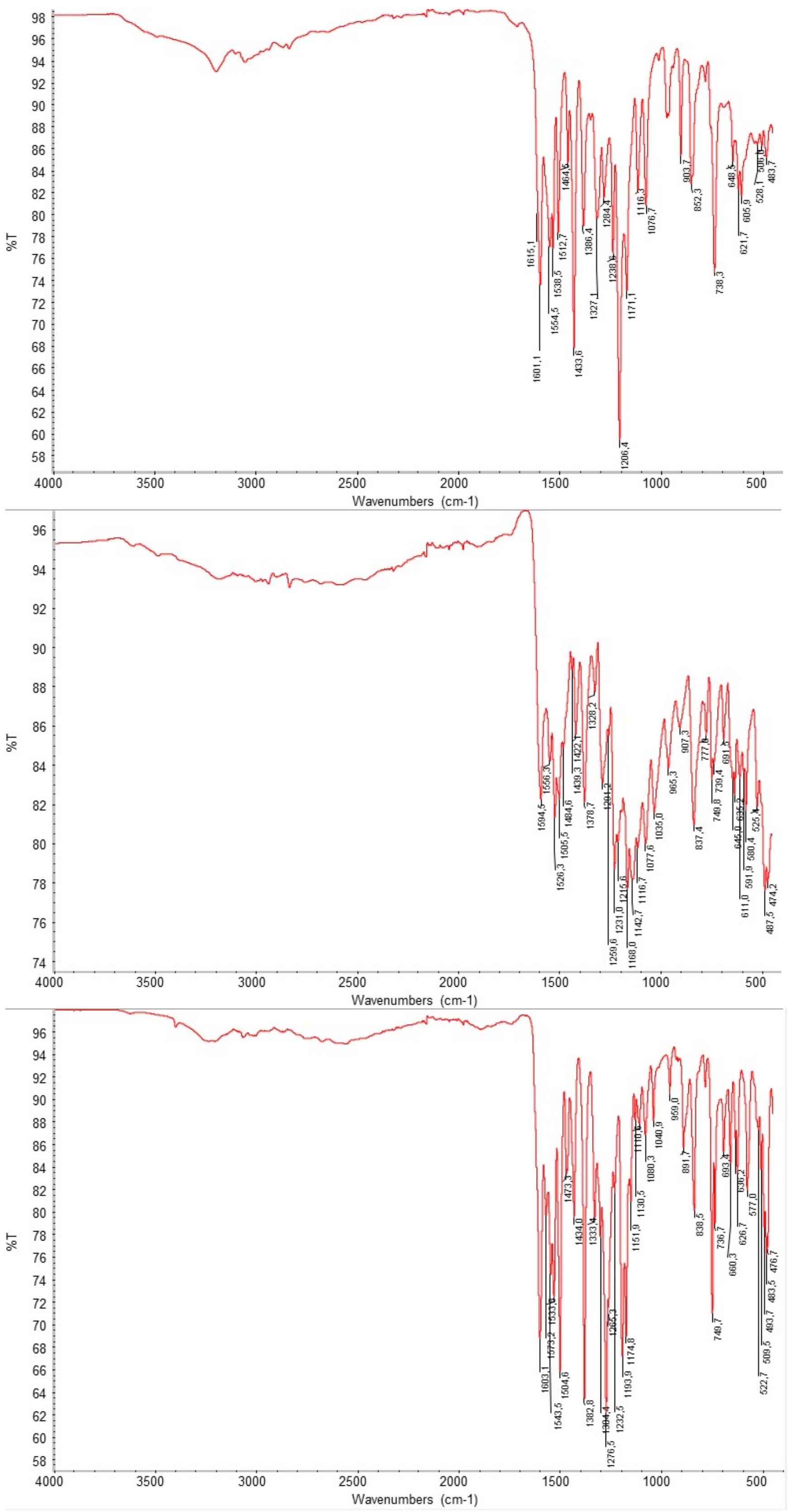

Figure S21 IR spectra of mononuclear nickel(II) complexes [Ni(HL)$_2$] (**7-9**) (from top to bottom).

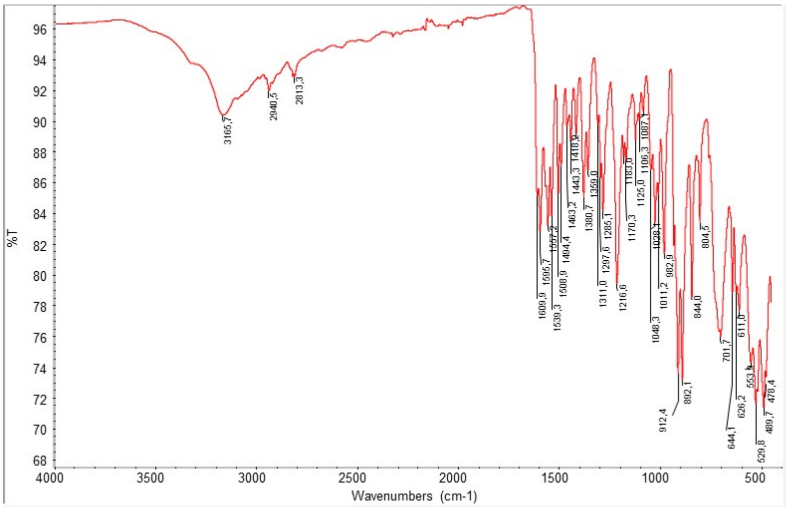

Figure S22 IR spectrum of hybrid organic-inorganic compound based on polyoxomolybdate $[Ni_2(HL^{4OMe})_2][Mo_4O_{10}(OCH_3)_6]$.

Ligands

Table S8. ^{1}H and ^{13}C chemical shifts (ppm) of compounds **H_2L^H**, **H_2L^{3OMe}**$\cdot H_2O$ and **H_2L^{4OMe}**$\cdot H_2O$

Atom	H_2L^H		$H_2L^{3OMe}\cdot H_2O$		$H_2L^{4OMe}\cdot H_2O$	
	δ / ppm (^{1}H)	δ/ppm (^{13}C)	δ / ppm (^{1}H)	δ / ppm (^{1}H)	δ / ppm (^{1}H)	δ / ppm (^{13}C)
**α**	8.63	147.72	8.64	147.59	8.55	148.21
**1**	–	118.66	–	118.88	–	111.81
**2**	–	157.45	–	147.18	–	159.41
**3**	6.91	119.23	–	147.90	6.52	106.29
**4**	7.30	131.08	7.12	121.02	–	161.88
**5**	6.94	116.37	6.87	118.92	6.53	106.29
**6**		131.08	7.02	113.72	7.39	131.17
**1'**	11.94	–	11.92	–	11.81	–
**2'**	–	162.48	–	162.46	–	162.31
**3'**	–	123.21	–	123.23	–	123.33
**4', 8'**	7.87	129.70	7.87	129.69	7.87	129.61
**5', 7'**	6.91	115.11	6.92	115.11	6.92	115.09
**6'**	10.18	160.91		160.89	–	160.81
**OH-2**	11.47	–	11.20	–	11.83	–
**OH-6'**	7.51	–	10.19	–	10.16	–
**OMe**	–	–	3.83	55.78	3.79	55.21

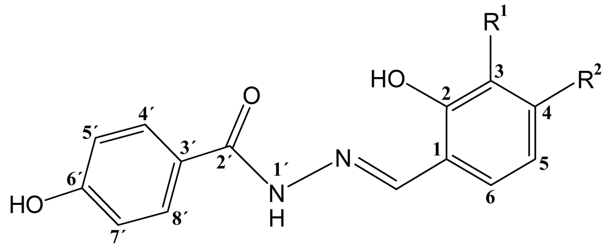

**R^1**	**R^2**	**ligand**
H	H	**H_2L^H**
OCH_3	H	**H_2L^{3OMe}**$\cdot H_2O$
H	OCH_3	**H_2L^{4OMe}**$\cdot H_2O$

Scheme S3 The structural formula of H_2L with the NMR numbering scheme
